# Matrix basis for plane and modal waves in a Timoshenko beam

**DOI:** 10.1098/rsos.160825

**Published:** 2016-11-30

**Authors:** Julio Cesar Ruiz Claeyssen, Daniela de Rosso Tolfo, Leticia Tonetto

**Affiliations:** 1 Instituto de Matemática, Universidade Federal do Rio Grande do Sul, 91.509-900 Porto Alegre, RS, Brazil; 2 Universidade Federal do Pampa, Campus Caçapava do Sul, 96.570-000, Caçapava do Sul, RS, Brazil; 3 Centro de Engenharias, Universidade Federal de Pelotas, 96.010-020, Pelotas, RS, Brazil

**Keywords:** plane waves, modal waves, Timoshenko beam, matrix basis, cracked beam, fundamental response

## Abstract

Plane waves and modal waves of the Timoshenko beam model are characterized in closed form by introducing robust matrix basis that behave according to the nature of frequency and wave or modal numbers. These new characterizations are given in terms of a finite number of coupling matrices and closed form generating scalar functions. Through Liouville’s technique, these latter are well behaved at critical or static situations. Eigenanalysis is formulated for exponential and modal waves. Modal waves are superposition of four plane waves, but there are plane waves that cannot be modal waves. Reflected and transmitted waves at an interface point are formulated in matrix terms, regardless of having a conservative or a dissipative situation. The matrix representation of modal waves is used in a crack problem for determining the reflected and transmitted matrices. Their euclidean norms are seen to be dominated by certain components at low and high frequencies. The matrix basis technique is also used with a non-local Timoshenko model and with the wave interaction with a boundary. The matrix basis allows to characterize reflected and transmitted waves in spectral and non-spectral form.

## 1. Introduction

In this paper, we present a matrix approach for characterizing all plane waves and modal waves of the Timoshenko beam. The wave matrix description is used in determining the reflection and transmission matrices for a crack problem.

Wave propagation and spectral analysis are two different methods for analysing the dynamic response of a structure. They are usually applied according to the duration of the temporal loading variation, structure size, experimental tests and discontinuities. The wave analysis uses the principle that an incident wave at certain point of interest will involve reflected and transmitted waves as particular cases of plane waves. The spectral method relates a time exponential behaviour with the spatial amplitude distribution to be determined according to involved boundary or compatibility conditions and thus leading to modal waves. Our matrix modal basis formulation could be eventually used in connection with the spectral finite-element method in the frequency domain or with the Fourier spectral method (SAM), whereas the plane basis formulation, which includes linear terms, could be eventually used when the spectral method uses conventional elements (SEM) in the spatial domain [1–5].

The Timoshenko equations constitute a distributed second-order evolutive system. When seeking plane waves or modal waves, we shall arrive at concentrated systems of second-order differential equations. These later can be studied by using a matrix basis generated by a closed-form fundamental response [6,7] that allows to obtain a wave matrix basis for describing all plane and modal waves of the Timoshenko beam. It will be shown that every modal wave can be decomposed as a linear superposition of plane waves. The shape of the waves is determined by a generating scalar function that appears in the closed form of the plane and modal matrix basis. The generating scalar function behaves quite well, regardless of varying parameters, that is, through limit procedures we can go from a dynamic situation to a static one [8]. Moreover, the modal-generating function is oscillatory above a critical frequency and evanescent below it.

In the literature, the unforced Timoshenko system is usually decoupled into two evolutive fourth-order differential equations that have the same form for the beam deflection and beam slope owing to bending [3,9] or it is assumed time periodic behaviour owing to the assumption of natural frequencies. These later assumptions lead to symmetric problems or to problems that behave in a periodic manner around cut-off frequencies and harmonic waves and normal modes have been the basic elements for their study. However, when the problem to be solved involves complicating effects such as damping, discontinuities, dispersive and complex materials, attached devices or obstacles, among others, harmonic behaviour or normal property is not often feasible [3]. Dispersion relations or characteristic equations can lead to real, pure imaginary or complex wavenumbers or temporal frequencies. It is thus convenient for general problems, from a mathematical point of view, to work out with plane waves or modal waves instead of classical harmonic progressive waves or to assume that the temporal behaviour is oscillating owing to natural frequencies and to have the normal mode property.

When a wave is incident upon some discontinuity in the beam, owing to geometric/material property change, kinetic constraints such as an elastic support or concentrated load, or boundary, it gives rise to reflected and transmitted waves whose characterization relate the amplitudes of incoming and outgoing waves at a discontinuity and could reveal local physical characteristics associated with structural vibrations of elastic media [10]. For a class of discontinuities found in applications, we can assume that they are described by elastic or dissipative or inertial forces that depend linearly with respect to spatial or time rates of deflection and rotation. Compatibility and boundary conditions can be formulated in matrix terms, so that by using wave propagation, we can have a systematic mathematical approach. This is illustrated with a crack problem where reflected and transmitted waves are identified from the matrix modal wave description and compatibility conditions.

The methodology developed in this work is new in the sense that it characterizes all plane and modal waves of the Timoshenko beam model without using the classical method of characteristics that leads to a coupled first-order system. Plane and modal waves of the Timoshenko model are obtained by solving in closed form complete second-order differential systems. Their behaviour can be studied in terms of associate scalar functions that change its geometrical form according to the nature of the complex frequency and wavenumber. Moreover, the matrix basis that characterizes plane and modal waves can be written as the superposition of exponential or linear terms.

The organization of this paper is presented as follows. The Timoshenko beam model is formulated as a second-order evolution matrix system and in decoupled form in §2. In §3, we characterize plane waves in matrix closed form depending upon a basis generated by a scalar solution of a fourth-order scalar differential equation. The cases of proportional components and plane waves of exponential type are discussed in detail. After that, a study of modal waves and its decomposition in plane waves is conducted in §4. A discussion about differences and relationships between plane and modal wave solutions is given in §5. Plane and modal waves for a non-local Timoshenko model are presented in §6 with the use of basis generated by fundamental matrix solutions of fourth- and second-order matrix differential equations, respectively. In §7, the methodology described in this paper is used in a crack problem. Boundary conditions are considered in §8. Finally, conclusions are drawn in §9.

## 2. Timoshenko model

The Timoshenko model for governing small amplitude transverse vibrations of a uniform beam with constant cross section [11] can be written in a Newtonian form as a second-order evolution equation that resembles a conservative model

2.1 $$\text{M}\ddot{\text{v}}(t,x)+\text{K}\text{v}(t,x)=\text{f},$$

where

2.2 $$\begin{array}{rl} \text{M} & =\left(\begin{array}{cc} \rho A & 0\\ 0 & \rho I \end{array}\right),\;\text{K}=\left(\begin{array}{cc} -\kappa GA\frac{\partial^{2}}{\partial x^{2}} & \kappa GA\frac{\partial }{\partial x}\\ -\kappa GA\frac{\partial }{\partial x} & -EI\frac{\partial^{2}}{\partial x^{2}}+\kappa GA \end{array}\right)\\ \;\mathrm{and}\;\;\text{v}(t,x) & =\left(\begin{array}{c} u(t,x)\\ \psi (t,x) \end{array}\right),\;\text{f}=\left(\begin{array}{c} f(t,x)\\ g(t,x) \end{array}\right) \end{array}\}$$

in which *A*,*E*,*G*,*I*,*κ*,*ρ*,*u*(*t*,*x*) and *ψ*(*t*,*x*) are cross-sectional area of the beam, the modulus of elasticity, shear modulus, cross-sectional moment of inertia, sectional shear coefficient, beam material density, beam deflection and beam slope owing to bending, respectively. The forcing terms *f* and *g* are the external distributed force load and moment, respectively.

The original formulation in terms of a second-order matrix differential equation is most suitable for the imposition of physically meaningful boundary or compatibility conditions. However, it is also possible to express the Timoshenko equations of motion for uniform beams in decoupled form when writing them in an algebraic form involving lambda matrices, that is,

2.3 Lv(t,x)=f,

with the matrix differential operator

$$\text{L}=\left(\begin{array}{cc} \rho A\frac{\partial^{2}}{\partial t^{2}}-\kappa GA\frac{\partial^{2}}{\partial x^{2}} & \kappa GA\frac{\partial }{\partial x}\\ -\kappa GA\frac{\partial }{\partial x} & \rho I\frac{\partial^{2}}{\partial t^{2}}-EI\frac{\partial^{2}}{\partial x^{2}}+\kappa GA \end{array}\right).$$

By applying Cramer’s rule det(**L**)**Iv**(*t*,*x*)=adj(**L**)**Lv**(*t*,*x*)=adj(**L**)**f** where adj(**L**) is the adjugate matrix of **L** and det(**L**) the determinant of **L**, we obtain the decoupled system

$$\begin{array}{rl} & \text{ce}\frac{\partial^{4}u(t,x)}{\partial t^{4}}+\left(\text{ca}-(\text{cb}+\text{ae})\frac{\partial^{2}}{\partial x^{2}}\right)\frac{\partial^{2}u(t,x)}{\partial t^{2}}+\text{ab}\frac{\partial^{4}u(t,x)}{\partial x^{4}}=F_{1}(t,x),\\ & \text{ce}\frac{\partial^{4}\psi (t,x)}{\partial t^{4}}+\left(\text{ca}-(\text{cb}+\text{ae})\frac{\partial^{2}}{\partial x^{2}}\right)\frac{\partial^{2}\psi (t,x)}{\partial t^{2}}+\text{ab}\frac{\partial^{4}\psi (t,x)}{\partial x^{4}}=F_{2}(t,x), \end{array}$$

with

$$F_{1}=e\frac{\partial^{2}f}{\partial t^{2}}-b\frac{\partial^{2}f}{\partial x^{2}}+af-a\frac{\partial g}{\partial x},\;F_{2}=c\frac{\partial^{2}g}{\partial t^{2}}+a\frac{\partial f}{\partial x}-a\frac{\partial^{2}g}{\partial x^{2}}$$

and

2.4 a=κGA,b=EI,c=ρA,e=ρI.

For a free homogeneous beam, we have *F*_1_=0 and *F*_2_=0 and the decoupling of the system leads to the study of the same fourth-order partial differential equation for the deflection and slope, that is,

2.5 $$\text{ce}\frac{\partial^{4}w}{\partial t^{4}}+\left(\text{ca}-(\text{cb}+\text{ae})\frac{\partial^{2}}{\partial x^{2}}\right)\frac{\partial^{2}w}{\partial t^{2}}+\text{ab}\frac{\partial^{4}w}{\partial x^{4}}=0.$$

Here, *w*(*t*,*x*) stands for the deflection *u*(*t*,*x*) or the slope o *ψ*(*t*,*x*). The hyperbolic character of (2.5) is discussed in [12]. This study is usually made with beams of infinite length. For beams of finite length where boundary conditions are imposed or multispan beams, there will an implicit coupling between deflection and slope, unless the conditions are separated as is the case with simply supported beams [11].

## 3. Plane waves of the Timoshenko beam model

Plane waves of the system (2.1) with *f*=0, *g*=0 are solutions of the type

3.1 u(t,x)=U(λt+βx),ψ(t,x)=Ψ(λt+βx),

where the scalars *β*=*μ*+*ik*, λ=*γ*+i*ω*, *c*=−λ/*β* are related to wavenumber, frequency and wave speed, respectively. By introducing the plane wave phase,

s=λt+βx,

we have that the wave profiles, *u*=U(*s*) and *ψ*=***Ψ***(*s*) are solutions of the system (2.1) whenever they satisfy the second-order differential system

$$\begin{array}{rl} & (\rho A\lambda^{2}-\kappa GA\beta^{2})U^{\prime \prime }(s)+\kappa GA\beta \Psi^{\prime }(s)=0,\\ & (\rho I\lambda^{2}-EI\beta^{2})\Psi^{\prime \prime }(s)-\kappa GA\beta U^{\prime }(s)+\kappa GA\Psi (s)=0. \end{array}$$

The above system can be written as a complete second-order matrix differential equation

3.2 $$M_{P}{\text{V}}^{\prime \prime }(s)+C_{P}{\text{V}}^{\prime }(s)+K_{P}\text{V}(s)=\text{0},$$

where

$$\begin{array}{rl} M_{P} & =\left(\begin{array}{cc} \rho A\lambda^{2}-\kappa GA\beta^{2} & 0\\ 0 & \rho I\lambda^{2}-EI\beta^{2} \end{array}\right),\;C_{P}=\left(\begin{array}{cc} 0 & \kappa GA\beta \\ -\kappa GA\beta & 0 \end{array}\right),\\ K_{P} & =\left(\begin{array}{cc} 0 & 0\\ 0 & \kappa GA \end{array}\right),\;\text{V}(s)=\left(\begin{array}{c} U(s)\\ \Psi (s) \end{array}\right). \end{array}$$

This matrix differential equation is regular when M_P_ is non-singular. The values of λ that make M_P_ singular are those for which

$$\det(M_{P})=(c\lambda^{2}-a\beta^{2})(e\lambda^{2}-b\beta^{2})=0,$$

that is

3.3 $$\lambda =\pm \sqrt{\frac{b}{e}}\beta ,\;\pm \sqrt{\frac{a}{c}}\beta .$$

In physical units, the above critical values (3.3) involve the rod and shear characteristic speeds $c_{R}=\sqrt{E/\rho },c_{S}=\sqrt{\kappa G/\rho }$, respectively [13]. For such values, we have the degenerated static case λ=0 and *β*=0 for which *u*=*u*_*o*_,*ψ*=0 is a constant solution.

From now on, we shall consider the regular case by assuming $\lambda \neq \pm \sqrt{E/\rho }\beta ,\pm \sqrt{\kappa G/\rho }\beta$. Thus, the spatial static case corresponds to λ=0 and *β*≠0, and the pure dynamic case corresponds to λ≠0 and *β*=0.

The general solution of equation (3.2) can be given in terms of a matrix basis that is generated by a fundamental matrix solution [6]. More precisely,

3.4 $$\text{V}(s)={\text{h}}_{P}(s)c_{1}+{\text{h}}_{P}^{\prime }(s)c_{2},$$

where **h**_P_(*s*) is the matrix solution of the initial value problem

$$\begin{array}{rl} & M_{P}{\text{h}}_{P}^{\prime \prime }(s)+C_{P}{\text{h}}_{P}^{\prime }(s)+K_{P}{\text{h}}_{P}(s)=\text{0},\\ & {\text{h}}_{P}(0)=\text{0},\;M_{P}{\text{h}}_{P}^{\prime }(0)=\text{I}. \end{array}$$

Here, **I** denotes the 2×2 identity matrix and **0** the 2×2 null matrix. The involved constants are arbitrary vectors c_1_=(*c*_11_ *c*_21_)^T^ and c_2_=(*c*_12_ *c*_22_)^T^. Moreover, the matrix solution **h**_P_(*s*) is given in closed form as

3.5 $${\text{h}}_{P}(s)=\left(\begin{array}{cc} \left(e\lambda^{2}-b\beta^{2})d_{P}^{\prime \prime }(s)+ad_{P}(s\right) & -a\beta d_{P}^{\prime }(s)\\ a\beta d_{P}^{\prime }(s) & (c\lambda^{2}-a\beta^{2})d_{P}^{\prime \prime } \end{array}\right),$$

where *d*_P_(*s*) is the solution of the scalar initial value problem

3.6 $$\begin{array}{rl} & b_{0}d_{P}^{\left(iv\right)}(s)+b_{2}d_{P}^{\prime \prime }(s)=0\\ \;\mathrm{and}\; & d_{P}(0)=0,\;d_{P}^{\prime }(0)=0,\;d_{P}^{\prime \prime }(0)=0,\;b_{0}d_{P}^{\prime \prime \prime }(0)=1, \end{array}\}$$

with *b*_0_,*b*_2_ being the coefficients of the polynomial

3.7 $$\begin{array}{rl} P(\eta ) & =\det(\eta^{2}M_{P}+\eta C_{P}+K_{P})=b_{0}\eta^{4}+b_{2}\eta^{2},\\ b_{0} & =ce\lambda^{4}-(cb+ae)\beta^{2}\lambda^{2}+ab\beta^{4}=(c\lambda^{2}-a\beta^{2})(e\lambda^{2}-b\beta^{2})\\ \;\mathrm{and}\;\;b_{2} & =ca\lambda^{2}, \end{array}\}$$

whose roots are

3.8 $$\begin{array}{rl} \eta & =0,0,\alpha ,-\alpha \\ \;\mathrm{and}\;\;\alpha & =\sqrt{-\frac{b_{2}}{b_{0}}}=\sqrt{\frac{-c\lambda^{2}a}{\left(c\lambda^{2}-a\beta^{2})(e\lambda^{2}-b\beta^{2}\right)}}. \end{array}\}$$

We observe that the root *α* is always well defined for the regular case once *b*_0_=(*c*λ^2^−*aβ*^2^)(*e*λ^2^−*bβ*^2^)≠0. Also, *α*=0 only when λ=0, with *β*≠0. In this case, the plane wave becomes a spatial permanent profile, and the root *η*=0 becomes quadruple.

By using the Laplace transform, it turns out that

3.9 $$d_{P}(s)=\frac{1}{ca\lambda^{2}}\left(s-\frac{\sinh(\alpha s)}{\alpha }\right)=\frac{1}{ca\lambda^{2}}\left(s-\frac{e^{\alpha s}-e^{-\alpha s}}{2\alpha }\right).$$

For λ=0, *β*≠0, we have *α*=0 and the plane waves have a permanent spatial profile. They can be obtained by the limit process

3.10 $$d_{P}(s)=\lim_{\lambda \to 0}\frac{1}{ca\lambda^{2}}\left(s-\frac{\sinh(\alpha s)}{\alpha }\right)=\frac{1}{6}\frac{s^{3}}{ab\beta^{4}}=\frac{1}{6}\frac{x^{3}}{ab\beta }.$$

Thus, we conclude that all plane waves of the classical Timoshenko model are characterized as

3.11 $$\text{v}(t,x)=\Phi_{P}(\lambda t+\beta x)c,$$

where

3.12 $$\begin{array}{rl} & \Phi_{P}(\lambda t+\beta x)=[{\text{h}}_{P}(\lambda t+\beta x)\;{\text{h}}_{P}^{\prime }(\lambda t+\beta x)]\\ & \;=\left(\begin{array}{cccc} (e\lambda^{2}-b\beta^{2})d_{P}^{\prime \prime }+ad_{P} & -a\beta d_{P}^{\prime } & (e\lambda^{2}-b\beta^{2})d_{P}^{\prime \prime \prime }+ad_{P}^{\prime } & -a\beta d_{P}^{\prime \prime }\\ a\beta d_{P}^{\prime } & (c\lambda^{2}-a\beta^{2})d_{P}^{\prime \prime } & a\beta d_{P}^{\prime \prime } & (c\lambda^{2}-a\beta^{2})d_{P}^{\prime \prime \prime } \end{array}\right), \end{array}$$

is a block matrix acting on the constant block vector

$$c=\left(\begin{array}{c} c_{1}\\ c_{2} \end{array}\right),\;c_{k}=\left(\begin{array}{c} c_{1k}\\ c_{2k} \end{array}\right),\;k=1,2.$$

Also, with *d*_P_(*s*) defined in (3.9), *s*=λ*t*+*βx*, we can also write Timoshenko plane waves as

3.13 $$\text{v}(t,x)=\Gamma_{P}(c,\lambda ,\beta )d_{P}(\lambda t+\beta x),$$

where

3.14 $$\begin{array}{rl} \Gamma_{P}(c,\lambda ,\beta ) & =\left(\begin{array}{cccc} ac_{11} & ac_{12}-a\beta c_{21} & (e\lambda^{2}-b\beta^{2})c_{11}-a\beta c_{22} & (e\lambda^{2}-b\beta^{2})c_{12}\\ 0 & a\beta c_{11} & a\beta c_{12}+(c\lambda^{2}-a\beta^{2})c_{21} & (c\lambda^{2}-a\beta^{2})c_{22} \end{array}\right)\\ \;\mathrm{and}\;d_{P}(\lambda t+\beta x) & =\left(\begin{array}{c} d_{P}(\lambda t+\beta x)\\ d_{P}^{\prime }(\lambda t+\beta x)\\ d_{P}^{\prime \prime }(\lambda t+\beta x)\\ d_{P}^{\prime \prime \prime }(\lambda t+\beta x) \end{array}\right). \end{array}\}$$

The pure dynamic case *β*=0, λ≠0 gives that *η*=0 is only a double root once

3.15 $$\alpha =\sqrt{-\frac{a}{e\lambda^{2}}}=\sqrt{\frac{-\kappa GA}{\rho I\lambda^{2}}}.$$

It is observed that when λ=λ_c_=i*ω*_c_, where

3.16 $$\omega_{c}=\sqrt{\frac{a}{e}}=\sqrt{\frac{\kappa GA}{\rho I}},$$

then *α*=1 and *ω*_c_ is a critical value that in spectral analysis is related to a cut-off or critical wave frequency [14]. In the next sections, we shall see that the value *α*=1 is related to plane waves of exponential form through a dispersion relation for λ and *β*.

**Figure 1.**
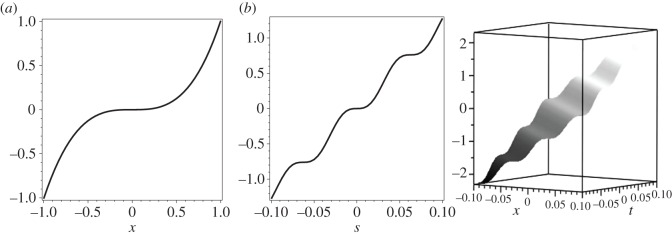
Profileof *d*_P_(*s*)=(1/*ca*λ^2^)(*s*−sinh(*αs*)/*α*), *s*=λ*t*+*βx*, for (*a*) λ=0, *β*=1 with *α*=0, (*b*) two- and three-dimensional profiles for λ=1, *β*=1, with *α*=100*i*.

From the above characterization of the Timoshenko plane waves, we have that there exist several types of plane waves **V**(λ*t*+*βx*) with complex λ=*γ*+i*ω* and *β*=*μ*+*ik*. Their nature depends upon the behaviour of the scalar-generating plane wave *d*_P_(*s*)=*d*_P_(λ*t*+*βx*) with respect to parameters λ, *β* and *α*. In figure 1, the solution *d*_P_(*s*) is illustrated for plane wave phase with different values of λ and *β* for an aluminium beam with rectangular cross section of height 2*h*=6×10^−6^ m, width *b*=3×10^−6^ m and parameters *E*=90 GPa, *ρ*=2700 kg m^−3^, *ν*=0.23 and moment of inertia *I*=2*bh*^3^/3 m^4^ given in [15]. The case (a) with λ=0, *β*=1 leads to the quadruple root *η*=0 once *α*=0 that is associated with the classical cubic deflection of a beam (3.10). The case (b) with λ=1 and *β*=1 leads to pure imaginary roots *η* once *α* will turn out a pure imaginary number. Thus, *d*_P_(*s*) will have an oscillating character with a linear trend.

### 3.1. Decomposition of plane waves

By using (3.9) in (3.5), Timoshenko plane waves can be decomposed in plane waves involving hyperbolic or exponential and linear functions. With the exponential formulation of *d*_P_(*s*) given in (3.9), we have

3.17 $$\Phi_{P}(s)=e^{\alpha s}{\text{L}}_{1}(\alpha )+e^{-\alpha s}{\text{L}}_{2}(\alpha )+s{\text{L}}_{3}+{\text{L}}_{4},$$

where L_*i*_, *i*=1,2,3,4, are the block matrices

3.18 $${\text{L}}_{1}(\alpha )=[A(\alpha )\;\alpha A(\alpha )],\;{\text{L}}_{2}(\alpha )={\text{L}}_{1}(-\alpha ),\;{\text{L}}_{3}=[B\;0],\;{\text{L}}_{4}=[C\;B]$$

with

$$\begin{array}{rl} A(\alpha ) & =\left(\begin{array}{cc} -\frac{1}{2}\frac{\alpha^{2}(e\lambda^{2}-b\beta^{2})+a}{ca\alpha \lambda^{2}} & \frac{1}{2}\frac{\beta }{c\lambda^{2}}\\ -\frac{1}{2}\frac{\beta }{c\lambda^{2}} & \frac{1}{2}\frac{(-c\lambda^{2}+a\beta^{2})\alpha }{ca\lambda^{2}} \end{array}\right),\\ B & =\left(\begin{array}{cc} \frac{1}{c\lambda^{2}} & 0\\ 0 & 0 \end{array}\right),\;C=\left(\begin{array}{cc} 0 & -\frac{\beta }{c\lambda^{2}}\\ \frac{\beta }{c\lambda^{2}} & 0 \end{array}\right), \end{array}$$

where *a*,*b*,*c*,*e* are given in (2.4). From (3.11)

$$\begin{array}{rl} \text{V}(s) & =\Phi_{P}(s)c=(A(\alpha )\;e^{\alpha s}+A(-\alpha )\;e^{-\alpha s}+sB+C)c_{1}\\ & \;+(\alpha A(\alpha )\;e^{\alpha s}-\alpha A(-\alpha )\;e^{-\alpha s}+B)c_{2}, \end{array}$$

and it follows the decomposition of plane waves

3.19 $$\begin{array}{rl} \text{v}(t,x) & =\Phi_{P}(\lambda t+\beta x)c\\ & =e^{\alpha (\lambda t+\beta x)}A(\alpha )(c_{1}+\alpha {\text{c}}_{2})+e^{-\alpha (\lambda t+\beta x)}A(-\alpha )(c_{1}-\alpha {\text{c}}_{2})\\ & \;+(\lambda t+\beta x)Bc_{1}+(Bc_{2}+Cc_{1}) \end{array}$$

for arbitrary vector constants c_1_ and c_2_. A similar procedure is followed when using hyperbolic functions.

### 3.2. Plane waves with proportional components

Plane waves propagating in one fixed direction, that is, their components are multiples of a scalar function *ϕ*(λ*t*+*βx*) can be completely characterized as those having an exponential or linear variation in certain directions. In fact, if **v**(*t*,*x*) is a solution of the Timoshenko model of the type

3.20 $$\text{v}(t,x)=\phi (\lambda t+\beta x)\left(\begin{array}{c} U_{o}\\ \Psi_{o} \end{array}\right)$$

their components must satisfy the fourth-order scalar differential equation given in (2.5). Thus,

3.21 $$b_{0}\phi^{\left(iv\right)}(s)+b_{2}\phi^{\prime \prime }(s)=0,$$

where *s*=λ*t*+*βx*, *b*_0_=*ce*λ^4^−(*cb*+*ae*)*β*^2^λ^2^+*abβ*^4^, *b*_2_=*ca*λ^2^ as in (3.7). Now, any solution of (3.21) can be written in terms of the basis generated by the solution *d*_P_(*s*) of the initial value problem (3.6) as *ϕ*(*s*)=*A*_1_*d*_P_(*s*)+*A*_2_*d*′_P_(*s*)+*A*_3_*d*_P_′′(*s*)+*A*_4_*d*_P_′′′(*s*). Then, *u*(*t*,*x*)=*ϕ*(λ*t*+*βx*)*U*_*o*_ and *ψ*(*t*,*x*)=*ϕ*(λ*t*+*βx*)*Ψ*_*o*_ will be the components of a solution of the Timoshenko equations (2.1), with **f**=**0**, whenever

3.22 $${\text{M}}_{\phi }\text{A}=\text{0},$$

where **M**_*ϕ*_ is the matrix

$$\left(\begin{array}{cccc} (-c\lambda^{2}+a\beta^{2})\alpha U_{o} & -a\beta \Psi_{o}\alpha & (-c\lambda^{2}+a\beta^{2})\alpha^{3}U_{o} & -a\beta \Psi_{o}\alpha^{3}\\ -a\beta \Psi_{o} & (-c\lambda^{2}+a\beta^{2})\alpha^{2}U_{o} & -a\beta \Psi_{o}\alpha^{2} & (-c\lambda^{2}+a\beta^{2})\alpha^{4}U_{o}\\ \beta \Psi_{o} & 0 & 0 & 0\\ (e\alpha^{2}\lambda^{2}-\alpha^{2}\beta^{2}b+a)\Psi_{o} & -\beta a\alpha^{2}U_{o} & (e\alpha^{2}\lambda^{2}-\alpha^{2}\beta^{2}b+a)\alpha^{2}\Psi_{o} & -\beta a\alpha^{4}U_{o}\\ -\beta a\alpha U_{o} & (e\alpha^{2}\lambda^{2}-\alpha^{2}\beta^{2}b+a)\alpha \Psi_{o} & -\beta a\alpha^{3}U_{o} & (e\alpha^{2}\lambda^{2}-\alpha^{2}\beta^{2}b+a)\alpha^{3}\Psi_{o}\\ \Psi_{o} & 0 & 0 & 0\\ \beta a\alpha U_{o} & -a\alpha \Psi_{o} & 0 & 0 \end{array}\right),$$

and A the vector with components *A*_1_, *A*_2_, *A*_3_, *A*_4_.

The above linear algebraic system has a non-zero solution only when *Ψ*_*o*_ vanishes or is conveniently chosen. The case *U*_*o*_=0 has only the trivial solution.

For *Ψ*_*o*_≠0, it is obvious that *A*_1_=0, *A*_2_=0. Thus, the system (3.22) reduces, after a Gaussian elimination, to

$$\left(\begin{array}{cc} (-c\lambda^{2}+a\beta^{2})\alpha^{3}U_{o} & -a\beta \Psi_{o}\alpha^{3}\\ 0 & \frac{-\alpha^{2}(2a\alpha^{2}\beta^{2}U_{o}^{2}c\lambda^{2}-a^{2}\alpha^{2}\beta^{4}U_{o}^{2}-c^{2}\alpha^{2}\lambda^{4}U_{o}^{2}+a^{2}\beta^{2}\Psi_{0}^{2})}{U_{o}(-c\lambda^{2}+a\beta^{2})} \end{array}\right)\left(\begin{array}{c} A_{3}\\ A_{4} \end{array}\right)=\left(\begin{array}{c} 0\\ 0 \end{array}\right).$$

By choosing *Ψ*_*o*_=±*αU*_*o*_(−*c*λ^2^+*aβ*^2^)/*aβ*, we shall have *A*_3_=±*αA*_4_, respectively.

Therefore,

$$\phi (s)=\pm \alpha A_{4}d_{P}^{\prime \prime }(s)+A_{4}d_{P}^{\prime \prime \prime }(s)=-A_{4}\frac{\alpha^{2}}{ac\lambda^{2}}\;e^{\pm \alpha s},$$

where *A*_4_ is an arbitrary constant.

For *Ψ*_*o*_=0 with *β*=0 or *β*≠0, the restrictions with the constants *A*_*i*_, *i*=1,…,4, in the system (3.22) imply that *ϕ*(*s*) is a linear function in *s*, homogeneous in *s* for *β*=0 and constant when *β*≠0. We conclude that all possible cases of having plane waves with proportional components of the type (3.20) are

$$\phi (s)=\left\{ \begin{array}{l} \frac{\alpha^{2}}{\text{ac}\lambda^{2}}\;e^{\pm \alpha s},\;\Psi_{o}=\pm \frac{\alpha U_{o}(-c\lambda^{2}+a\beta^{2})}{a\beta }\neq 0\\ -\frac{\alpha^{2}}{\left(c\lambda^{2}a\right)}(A_{3}s+A_{4}),\;\Psi_{o}=0,\beta =0\\ 1,\;\Psi_{o}=0,\;\beta \neq 0 \end{array}\right.,$$

and the waves propagate in the directions

$$\left(\begin{array}{c} 1\\ \frac{\pm \alpha (-c\lambda^{2}+a\beta^{2})}{a\beta } \end{array}\right),\;\left(\begin{array}{c} 1\\ 0 \end{array}\right),$$

with exponential and linear variation, respectively.

### 3.3. Waves with exponential profile

For plane waves, the relation between the complex scalars,

λ=γ+iω,β=μ+ik,

is somehow arbitrary. However, the search of plane waves that have an exponential profile

3.23 $$u(t,x)=e^{\lambda t+\beta x}U_{0},\;\psi (t,x)=e^{\lambda t+\beta x}\Psi_{0},$$

will relate λ and *β* through algebraic equation. When λ=i*ω* and *β*=i*k* are pure imaginary, the wave phase *θ*=*kx*+*ωt* is 2*π* periodic in time and space. The value λ=i*ω* associated with temporal frequency or simply frequency and *β*=i*k* associated with the wavenumber *k* or wavelength 2*π*/*k* [10].

When applying wave mechanics to a beam of finite length *L*, it is understood that the beam is considered to repeat per unit distance in order to have a periodic position in space or to be considered a beam of infinite length. It should be observed that for pinned–pinned beams, it turns out that the spatial periodicity can be assumed to be a natural one. For other kind of classical boundary conditions, we can still have frequencies λ=i*ω*, *ω* real, or assume harmonic wave motion in order to apply the spectral method.

However, this is not the case when dealing with non-classical boundary conditions or with beams made of new materials that are used to improve and optimize its properties and subjected to internal and external damping [16–18].

By substitution of (3.23) in the Timoshenko system (2.1), it turns out that we have to determine non-zero solutions of the algebraic system

3.24 $$\left(\begin{array}{cc} c\lambda^{2}-a\beta^{2} & a\beta \\ -a\beta & e\lambda^{2}-b\beta^{2}+a \end{array}\right)\left(\begin{array}{c} U_{o}\\ \Psi_{o} \end{array}\right)=\left(\begin{array}{c} 0\\ 0 \end{array}\right),$$

where *a*=*κGA*, *b*=*EI*, *c*=*ρA*, *e*=*ρI*. To ensure the existence of non-zero solutions, the determinant of the system must be zero. Thus, λ and *β* must satisfy the equation

3.25 $$Q(\lambda ,\beta )=ce\lambda^{4}-(cb+ae)\beta^{2}\lambda^{2}+ca\lambda^{2}+ab\beta^{4}=0$$

that is referred to in the literature as the dispersion equation. When *Q*(λ,*β*)=*b*_0_+*b*_2_=0, we have that *η*=*α*=1 is a root of *P*(*η*). This equation is the same when seeking scalar plane exponential solutions in (2.5). Moreover, when solving for λ=λ(*β*) or *β*=*β*(λ), they will appear in pairs, that is,

3.26 $$\pm \beta_{1},\;\pm \beta_{2},\;\pm \lambda_{1},\;\pm \lambda_{2}.$$

We obtain from (3.24)

3.27 $$\left(\begin{array}{c} U_{o}\\ \Psi_{o} \end{array}\right)=\Psi_{o}\left(\begin{array}{c} \xi \\ 1 \end{array}\right)=\left(\begin{array}{c} \Psi_{o}\xi \\ \Psi_{o} \end{array}\right),\;\xi =-\frac{a\beta }{c\lambda^{2}-a\beta^{2}}.$$

The denominator of *ξ* is always not zero for we are not considering the singular case (3.3). Thus, the wave exponential will be a multiple of the basic solution

3.28 $$\text{v}(t,x)=e^{\lambda t+\beta x}\left(\begin{array}{c} \xi \\ 1 \end{array}\right).$$

When *β*=0, we have the exponential plane wave *u*=e^λ_c_*t*^*U*_*o*_,*ψ*=e^λ_c_*t*^*Ψ*_*o*_ with

3.29 $$\left(\begin{array}{c} U_{o}\\ \Psi_{o} \end{array}\right)=\Psi_{o}\left(\begin{array}{c} 0\\ 1 \end{array}\right).$$

The movement is referred to as a shearing motion or pure dynamic case (3.15), (3.16) corresponding to the critical value λ_c_=i*ω*_c_.

#### 3.3.1. Exponential waves as plane waves

The basic exponential wave solution obtained from (3.23) and (3.27)

3.30 $$\text{v}(t,x)=e^{\lambda t+\beta x}\left(\begin{array}{c} \xi \\ 1 \end{array}\right),\;\xi =-\frac{a\beta }{c\lambda^{2}-a\beta^{2}}$$

can be written in terms of the decomposition of plane waves (3.19) as

3.31 $$\text{v}(t,x)=e^{\lambda t+\beta x}\left(\begin{array}{c} \xi \\ 1 \end{array}\right)=\Phi_{P}(\lambda t+\beta x)\text{c}(\lambda ,\beta ),\;\text{c}(\lambda ,\beta )=\left(\begin{array}{c} 0\\ \frac{ab_{2}}{b_{0}}\\ \frac{ab_{2}\beta }{b_{0}}\\ \frac{b_{2}}{-c\lambda^{2}+a\beta^{2}} \end{array}\right),$$

where *b*_0_,*b*_2_ are as in (3.7) and *α*=1 once *β* and λ will satisfy *Q*(λ,*β*)=0. For *β*=0 a regular value, we have λ=i*ω*_c_, *ω*_c_=*a*/*e* and *ξ*=0.

For a fixed λ, a general exponential-type wave solution will be obtained by superposition with all the roots *β* of the dispersion equation (3.25), that is

3.32 $$\begin{array}{rl} \text{v}(t,x) & =p_{1}\Phi_{P}(\lambda t+\beta_{1}x)\text{c}(\lambda ,\beta_{1})+p_{2}\Phi_{P}(\lambda t-\beta_{1}x)\text{c}(\lambda ,-\beta_{1})\\ & \;+p_{3}\Phi_{P}(\lambda t+\beta_{2}x)\text{c}(\lambda ,\beta_{2})+p_{4}\Phi_{P}(\lambda t-\beta_{2}x)\text{c}(\lambda ,-\beta_{2}), \end{array}$$

where *p*_1_,*p*_2_,*p*_3_,*p*_4_ are arbitrary scalars.

## 4. Modal waves

Time exponential solutions with spatial dependence varying amplitude of the Timoshenko model will be called modal waves. In matrix terms, these latter waves are solutions of the homogeneous Timoshenko model $\text{M}\ddot{\text{v}}+\text{Kv}=\text{0}$ of the form

4.1 $$\text{v}(t,x)=e^{\lambda t}\text{w}(x),\;\text{w}(x)=\left(\begin{array}{c} U(x)\\ \Psi (x) \end{array}\right),$$

where λ is an arbitrary but fixed scalar. They arise in modal analysis for vibrating problems where we seek to find natural frequencies for beams of finite length subject to classical boundary conditions such as the simply supported or fixed-pinned cases, among others. For such conditions, it is well known that λ=i*ω* is always pure imaginary and the spatial amplitudes **w**(*x*) corresponding to different values of the natural frequency *ω* are orthogonal [11]. In physics, they are referred to as standing or stationary waves. We shall prove below that they are superposition of plane waves.

Modal waves will exist for the unforced Timoshenko model (2.1), provided **w**(*x*) satisfies the second-order matrix differential equation

4.2 $$M{\text{w}}^{\prime \prime }(x)+C{\text{w}}^{\prime }(x)+K(\lambda )\text{w}(x)=\text{0},$$

where

$$M=\left(\begin{array}{cc} -\kappa GA & 0\\ 0 & -EI \end{array}\right),\;C=\left(\begin{array}{cc} 0 & \kappa GA\\ -\kappa GA & 0 \end{array}\right),\;K=\left(\begin{array}{cc} \rho A\lambda^{2} & 0\\ 0 & \rho I\lambda^{2}+\kappa GA \end{array}\right).$$

The solution **w**(*x*) is called eigenfunction or vibration mode corresponding to the eigenvalue λ, that in the frequency domain is denoted by **w**(λ,*x*).

The general solution of equation (4.2) is given by

4.3 $$\text{w}(x)=\text{h}(x)a_{1}+\text{h'}(x)a_{2},$$

where **h**(*x*) is 2×2 matrix solution satisfying

4.4 $$\begin{array}{rl} & M{\text{h}}^{\prime \prime }(x)+C{\text{h}}^{\prime }(x)+K(\lambda )\text{h}(x)=\text{0}\\ \;\mathrm{and}\; & \text{h}(0)=\text{0},\;M{\text{h}}^{\prime }(0)=\text{I}, \end{array}\}$$

with **0** the 2×2 null matrix, **I** the 2×2 identity matrix and

4.5 $$a=\left(\begin{array}{c} a_{1}\\ a_{2} \end{array}\right),\;a_{1}=\left(\begin{array}{c} a_{11}\\ a_{21} \end{array}\right),\;a_{2}=\left(\begin{array}{c} a_{12}\\ a_{22} \end{array}\right).$$

We have that [7]

4.6 $$\text{h}(x)=\left(\begin{array}{cc} -bd^{\prime \prime }(x)+(a+e\lambda^{2})d(x) & -ad^{\prime }(x)\\ ad^{\prime }(x) & -ad^{\prime \prime }(x)+c\lambda^{2}d(x) \end{array}\right),$$

where *d*(*x*) is solution of the initial value problem

4.7 $$\begin{array}{rl} & b_{0}d^{\left(iv\right)}(x)+b_{2}d^{\prime \prime }(x)+b_{4}d(x)=0\\ \;\mathrm{and}\; & d(0)=0,\;d^{\prime }(0)=0,\;d^{\prime \prime }(0)=0,\;b_{0}d^{\prime \prime \prime }(0)=1, \end{array}\}$$

where $Q(z)=\det(z^{2}M+zC+K)=b_{0}z^{4}+b_{2}z^{2}+b_{4}$ with *b*_0_=*ab*, *b*_2_=−(*ae*+*bc*)λ^2^ and *b*_4_=(*e*λ^2^+*a*)*c*λ^2^.

In matrix terms, we have that all modal waves are of the type

4.8 $$\text{v}(t,x)=e^{\lambda t}\Phi_{M}(x)a=e^{\lambda t}\Gamma_{M}(a,\lambda ,\beta )d_{M}(x),$$

where

4.9 $$\Phi_{M}(x)=\left(\begin{array}{cccc} -bd^{\prime \prime }(x)+(a+e\lambda^{2})d(x) & -ad^{\prime }(x) & -bd^{\prime \prime \prime }(x)+(a+e\lambda^{2})d^{\prime }(x) & -ad^{\prime \prime }(x)\\ ad^{\prime }(x) & -ad^{\prime \prime }(x)+c\lambda^{2}d(x) & ad^{\prime \prime }(x) & -ad^{\prime \prime \prime }(x)+c\lambda^{2}d^{\prime }(x) \end{array}\right)$$

is a modal matrix basis,

4.10 $$\Gamma_{M}(a,\lambda ,\beta )=\left(\begin{array}{cccc} (a+e\lambda^{2})a_{11} & -aa_{21}+(a+e\lambda^{2})a_{12} & -ba_{11}-aa_{22} & -ba_{12}\\ \lambda^{2}ca_{21} & aa_{11}+\lambda^{2}ca_{22} & -aa_{21}+aa_{12} & -aa_{22} \end{array}\right)$$

and

4.11 $$d_{M}(x)=\left(\begin{array}{c} d(x)\\ d^{\prime }(x)\\ d^{\prime \prime }(x)\\ d^{\prime \prime \prime }(x) \end{array}\right).$$

The solution *d*(*x*) can be found by using Laplace transform in (4.7). It turns out that

4.12 $$d(x)=L^{-1}(H(z)),\;H(z)=\frac{1}{Q(z)},$$

where

4.13 $$Q(z)=\det(z^{2}M+zC+K)=abz^{4}-(ae+bc)\lambda^{2}z^{2}+(a+e\lambda^{2})c\lambda^{2}$$

is just the polynomial value *P*(1)=*b*_0_+*b*_2_ given in (3.7) with *β*=*z* and λ arbitrary but fixed. The roots *β* of (4.13) are called modal numbers.

Let *z*=±*β*_1_(λ), ±*β*_2_(λ) be the roots of the characteristic polynomial (4.13). Then, for the case of simple roots, we obtain that

4.14 $$d(x)=\sum_{k=1}^{2}\left[\frac{e^{\pm \beta_{k}x}}{P^{\prime }(\pm \beta_{k})}\right]=\frac{\beta_{2}\sinh(\beta_{1}x)-\beta_{1}\sinh(\beta_{2}x)}{ab(\beta_{1}^{2}-\beta_{2}^{2})\beta_{1}\beta_{2}}.$$

Double roots of the polynomial (4.13) occur only when λ assume the values $\lambda_{c}=\sqrt{-a/e}$ (*z*_c_=0) and $\lambda_{a}=(2a\sqrt{bc}/\left(ae-bc\right))(z_{a}^{2}=(\left(ae+bc\right)/2ab)\lambda_{a}^{2}\neq 0$). We have that *z*=0 is the unique quadruple root and occurs only when λ=0. There are no other kind of repeated roots.

For λ=λ_c_, we have that *β*_1_=0 or *β*_2_=0 cannot both be double roots. In what follows, we shall denote *β*_1_=0 to be the double root. We can use partial fractions for 1/*P*(*s*) or take limit when $\beta_{1}\to 0$ to obtain *d*(*x*). With this later process, it follows that

4.15 $$d(x)=\lim_{\beta_{1}\to 0}\frac{\beta_{2}\sinh(\beta_{1}x)-\beta_{1}\sinh(\beta_{2}x)}{ab(\beta_{1}^{2}-\beta_{2}^{2})\beta_{1}\beta_{2}}=\frac{\sinh(\beta_{2}x)-\beta_{2}x}{\beta_{2}^{3}ab}.$$

When *β*_1_=*β*_2_=0, we have a quadruple root and repeated, use of the L’Hospital rule gives us

$$d(x)=\lim_{\beta_{2}\to 0,\beta_{1}\to 0}\frac{\beta_{2}\sinh(\beta_{1}x)-\beta_{1}\sinh(\beta_{2}x)}{ab(\beta_{1}^{2}-\beta_{2}^{2})\beta_{1}\beta_{2}}=\frac{1}{6}\frac{x^{3}}{ab}.$$

For λ=±λ_*a*_, $\lambda_{a}=2a\sqrt{cb}/\left(ae-cb\right)$, we have a couple of double roots ±*β*_*a*_, where $\beta_{a}^{2}=(\left(cb+ae\right)/2ab)\lambda_{a}^{2}$. In this case, the characteristic polynomial given in (4.13) can be written as

$$Q=ab{\left(z-\beta_{a}\right)}^{2}{\left(z+\beta_{a}\right)}^{2},$$

and by Laplace transform we have

4.16 $$d(x)=\frac{\cosh(\beta_{a}x)x\beta_{a}-\sinh(\beta_{a}x)}{ab\beta_{a}^{3}}.$$

### 4.1. Natural frequencies and roots

The polynomial *Q*(*z*) defined in (4.13), that arises in connection with modal waves for the Timoshenko equations, can be conveniently written as

4.17 $$z^{4}+g^{2}(\lambda )z^{2}-r^{4}(\lambda )=0,$$

where

$$g^{2}(\lambda )=-\left(\frac{e}{b}+\frac{c}{a}\right)\lambda^{2},\;r^{4}(\lambda )=-c\lambda^{2}\left(\frac{a+e\lambda^{2}}{ab}\right).$$

The roots of (4.17) are *z*=±*ϵ* and *z*=±i*δ*, where

4.18 $$\epsilon =\frac{1}{2}\sqrt{-2g^{2}+2\sqrt{\Omega }},\;\delta =\frac{1}{2}\sqrt{2g^{2}+2\sqrt{\Omega }},\;\Omega =g^{4}+4r^{4}.$$

The roots *z*=±*ϵ*,±i*δ* are related by the equation

4.19 $$\delta^{2}-\epsilon^{2}=g^{2}.$$

By substituting *β*_1_=*ϵ* and *β*_2_=i*δ* in (4.14), the fundamental solution *d*(*x*) can be written

4.20 $$d(x)=\frac{\delta \sinh(\epsilon x)-\epsilon \sin(\delta x)}{ab(\delta^{2}+\epsilon^{2})\epsilon \delta }.$$

When λ is a natural frequency, the roots *ϵ* and i*δ* can be identified in terms of the critical or cut-off frequency $\omega_{c}=\sqrt{a/e}$ introduced in (3.16). By substituting λ=i*ω*, *ω*^2^>0, in *Ω* (4.18), it turns out that the value *δ* is always real positive for all *ω* non-zero real once

$$\Omega ={\left(\frac{e}{b}-\frac{c}{a}\right)}^{2}\omega^{4}+4\omega^{2}\frac{c}{b}>0.$$

The nature of *ϵ* depends on the value *ω* being below or above the frequency *ω*_c_. For *ω*^2^<*ω*^2^_c_, the value of *ϵ* is real and non-zero once *r*^4^>0. We have *ϵ*=0 when *ω*=*ω*_c_ once for this value we have *r*^4^=0. For *ω*^2^>*ω*^2^_c_, it follows that *r*^4^<0 and, consequently, *ϵ*^2^<0. This later gives *ϵ*=i*ε* with *ε*>0.

Therefore, above the critical frequency, we have the dispersion equation for harmonic propagating waves

4.21 $$Q(i\omega ,ik)=abk^{4}-(ae+bc)\omega^{2}k^{2}-ac\omega^{2}+ce\omega^{4}=0,$$

with *k* being a real wavenumber and (4.20) being now the oscillatory solution

4.22 $$d(x)=\frac{\delta \sin(\varepsilon x)-\varepsilon \sin(\delta x)}{ab(\delta^{2}-\varepsilon^{2})\varepsilon \delta }.$$

There are several types of modal waves e^λ*t*^**w**(*x*) according to the nature of the spatial amplitude **w**(*x*) and linear superposition. From (4.8) and (4.14), the behaviour will depend on the nature of the roots ±*β*_1_(λ),±*β*_2_(λ) of the polynomial

4.23 $$abz^{4}-(ae+bc)\lambda^{2}z^{2}+(a+e\lambda^{2})c\lambda^{2}=0,$$

where λ is an arbitrary but fixed scalar (3.25).

**Figure 2.**
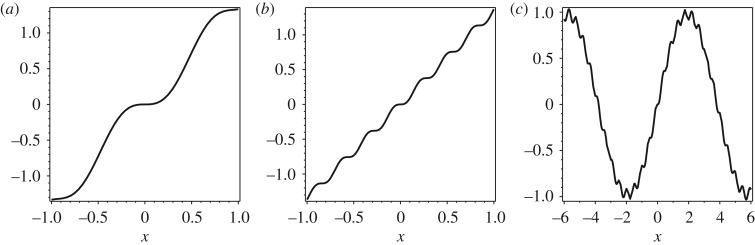
The scalar-generating modal waves $d(x)=(\delta \sinh(\epsilon x)-\epsilon \sin(\delta x))/ab(\delta^{2}+\epsilon^{2})\epsilon \delta ,$ where (*a*) *ϵ*=42735.46244, *δ*= 6.649479825×10^5^ for $\omega^{2}<\omega_{c}^{2}$; (*b*) *ϵ*=0, *δ*=6.707335165×10^5^ for *ω*^2^=*ω*^2^_c_; (*c*) *ϵ*= 40931.10798*i*, *δ*=6.759469095×10^5^ for *ω*^2^>*ω*^2^_c_.

By considering the parameters for an aluminium microbeam given in [15], we can observe that the behaviour of *d*(*x*) in (4.20) depends upon the natural frequency *ω* in relation to the critical frequency *ω*_c_. For *ω*^2^<*ω*^2^_c_, the characteristic equation (4.13) has two simple reals roots ±*β*_1_=±*ϵ* and two simple conjugate pure imaginary roots ±*β*_2_=±i*δ*. This implies that *d*(*x*) is composed of hyperbolic and trigonometric functions whose behaviour is illustrated in figure 2*a*. For *ω*=*ω*_c_, the real roots of (4.13) collapse and become the double root ±*β*_1_=0 and remain the simple conjugate pure imaginary roots ±*β*_2_=±i*δ*. The function *d*(*x*) is now composed of linear and trigonometric functions and whose unbounded oscillating behaviour is presented in figure 2*b*. When above the critical frequency, *ω*^2^>*ω*^2^_c_, the four roots of (4.13) are two pairs of simple conjugate pure imaginary. Thus, *d*(*x*) is composed of trigonometric functions with a bounded oscillatory behaviour as observed in figure 2*c* having ripples of high-frequency content.

### 4.2. The critical case: Liouville technique

The value *β*=0 is defective once the system (3.24) has only a single independent solution. For the critical value $\lambda =\sqrt{-a/e}$, which corresponds to *β*_1_=0 being a double root of the polynomial *Q*(λ,*β*) defined in (3.25), we need to find a second linearly independent solution associated with such *β*_1_. No modification is needed for *β*_2_ because it is non-zero, otherwise, *β*=0 will be a quadruple root.

In the literature, in order to find another independent solution, we can use mathematical techniques for repeated roots or use physical arguments as in [19] once from (3.30) we have that ξ→0 as β→0. Here, we shall use the Liouville technique of differentiating a plane wave with respect to a parameter that in a limit, assumes the same value of another one.

Thus, by differentiating (3.13) with respect to *β* when $\lambda_{c}=\sqrt{-\frac{a}{e}}$ and *α*=1, we obtain

$$\frac{\partial \text{v}(t,x)}{\partial \beta }=\frac{\partial \Gamma_{P}}{\partial \beta }\text{d}(\lambda t+\beta x)+\Gamma_{P}{\frac{\partial d(\lambda t+\beta x)}{\partial \beta }|}_{\beta =0}=\left(\begin{array}{c} a_{1}\\ a_{1}\frac{c}{e}x+a_{2} \end{array}\right)\sinh(\lambda_{c}t),$$

where *a*_1_,*a*_2_ are arbitrary constants. Thus

$$\text{v}(t,x)=\left(\begin{array}{c} 1\\ \frac{c}{e}x \end{array}\right)\sinh(\lambda_{c}t),\;\lambda_{c}=\sqrt{-a/e}$$

is a second linearly independent solution. We also observe that by superposition of plane wave solutions (3.13) and convenient constants, we get

4.24 $$\text{v}(t,x)=\left(A_{1}\left(\begin{array}{c} 1\\ \frac{c}{e}x \end{array}\right)+A_{2}\left(\begin{array}{c} 0\\ 1 \end{array}\right)\right)e^{\sqrt{-(a/e)t}},$$

where *A*_1_ and *A*_2_ are arbitrary constants.

The double root case *β*_1_=0 with λ=λ_c_ was treated above with exponential waves. When using the modal wave formulation, such solution can be obtained by using the same Liouville technique that leads to *d*(*x*) given in (4.15). Thus for the critical case λ=i*ω*_c_, we have

4.25 $$\text{v}(t,x)=e^{\lambda_{c}t}\left(\left(\begin{array}{c} A_{1}\cosh(\beta_{2}x)\\ A_{2}\sinh(\beta_{2}x) \end{array}\right)+\left(\begin{array}{c} A_{3}\sinh(\beta_{2}x)\\ A_{4}\cosh(\beta_{2}x) \end{array}\right)+\left(\begin{array}{c} A_{5}\\ A_{6}x \end{array}\right)+\left(\begin{array}{c} 0\\ A_{7} \end{array}\right)\right),$$

where *A*_*i*_=*A*_*i*_(*a*_11_,*a*_12_,*a*_21_,*a*_22_) can be chosen in terms of the arbitrary constants *a*_11_,*a*_12_,*a*_21_,*a*_22_, [20].

The same arguments apply to the case λ=λ_*a*_ for which there are two pairs ±*β*_*a*_ of double roots once the Liouville technique used with such values also provides a solution of the Timoshenko equations. In this situation, we use *d*(*x*) given in (4.16).

The relation with the exponential basis

4.26 $$\left(\begin{array}{c} d(x)\\ d^{\prime }(x)\\ d^{\prime \prime }(x)\\ d^{\prime \prime \prime }(x) \end{array}\right)=\frac{1}{2ab(\beta_{1}^{2}-\beta_{2}^{2})}\left(\begin{array}{cccc} \frac{1}{\beta_{1}} & -\frac{1}{\beta_{1}} & -\frac{1}{\beta_{2}} & \frac{1}{\beta_{2}}\\ 1 & 1 & -1 & -1\\ \beta_{1} & -\beta_{1} & -\beta_{2} & \beta_{2}\\ \beta_{1}^{2} & \beta_{1}^{2} & -\beta_{2}^{2} & -\beta_{2}^{2} \end{array}\right)\left(\begin{array}{c} e^{\beta_{1}x}\\ e^{-\beta_{1}x}\\ e^{\beta_{2}x}\\ e^{-\beta_{2}x} \end{array}\right),$$

can be written in compact form

4.27 $${\text{d}}_{\text{M}}(x)={\text{M}}_{d}^{\exp}\;\mathrm{Exp}(x),$$

where

4.28 $${\text{M}}_{d}^{\exp}=\frac{1}{2ab(\beta_{1}^{2}-\beta_{2}^{2})}\left(\begin{array}{cccc} \frac{1}{\beta_{1}} & -\frac{1}{\beta_{1}} & -\frac{1}{\beta_{2}} & \frac{1}{\beta_{2}}\\ 1 & 1 & -1 & -1\\ \beta_{1} & -\beta_{1} & -\beta_{2} & \beta_{2}\\ \beta_{1}^{2} & \beta_{1}^{2} & -\beta_{2}^{2} & -\beta_{2}^{2} \end{array}\right),\;\mathrm{Exp}(x)=\left(\begin{array}{c} e^{\beta_{1}x}\\ e^{-\beta_{1}x}\\ e^{\beta_{2}x}\\ e^{-\beta_{2}x} \end{array}\right).$$

The case of the repeated root *β*_1_=0 is worked from above as a limit procedure.

### 4.3. Decomposition of modal waves

By using (4.14) in *Φ*_*M*_(*x*), we can write

4.29 $$\Phi_{M}(x)=e^{\beta_{1}x}\Psi_{1}(\beta_{1})+e^{\beta_{2}x}\Psi_{2}(\beta_{2})+e^{-\beta_{1}x}\Psi_{1}(-\beta_{1})+e^{-\beta_{2}x}\Psi_{2}(-\beta_{2}),$$

where *Ψ*_1_(*β*_1_),*Ψ*_2_(*β*_2_) are the block matrices

4.30 $$\Psi_{1}(\beta )=\Psi (\beta ),\;\Psi_{2}(\beta )=-\Psi (\beta )$$

with

$$\Psi (\beta )=[A_{M}(\beta )\;\beta A_{M}(\beta )],\;A_{M}(\beta )=\left(\begin{array}{cc} \frac{1}{2}\frac{e\lambda^{2}-b\beta^{2}+a}{ab(\beta_{1}^{2}-\beta_{2}^{2})\beta } & -\frac{1}{2b(\beta_{1}^{2}-\beta_{2}^{2})}\\ \frac{1}{2b(\beta_{1}^{2}-\beta_{2}^{2})} & \frac{1}{2}\frac{c\lambda^{2}-a\beta^{2}}{ab(\beta_{1}^{2}-\beta_{2}^{2})\beta } \end{array}\right),$$

where *a*,*b*,*c*,*e* are given in (2.4). Thus

4.31 $$\begin{array}{rl} \text{v}(t,x) & =e^{\lambda t}\Phi_{M}(x)a=e^{\lambda t+\beta_{1}x}\text{ v}(\beta_{1})-e^{\lambda t+\beta_{2}x}\text{v}(\beta_{2})\\ & \;+e^{\lambda t-\beta_{1}x}\text{ v}(-\beta_{1})-e^{\lambda t-\beta_{2}x}\text{v}(-\beta_{2}), \end{array}$$

where

4.32 $$\text{v}(\beta )=A_{M}(\beta )(a_{1}+\beta a_{2}).$$

The above decomposition in exponential waves is also valid for the critical value by taking limit when *β*_1_ approaches 0 as long as we do not cancel zero divisors.

The spectral superposition in [19] can be obtained from above by observing that the eigenvectors $V(\xi )=\left(\begin{array}{c} \xi (\beta )\\ 1 \end{array}\right)$ given in (3.30), are multiples of the vectors **v**(*β*). With the second component of **v**(*β*) and use of (3.32) and (4.13), we can set up a system **P**a=c in order to determine the constant vector a. For instance, by choosing in c the first component equal to 1 and zero the others components, we obtain the eigenvector *V* (*ξ*_1_). In general, by defining

$$a(\gamma ,\eta )=\left(\begin{array}{c} \frac{(c\lambda^{2}-a\eta^{2})b}{a}\\ -\frac{ab\gamma \eta^{2}}{c\lambda^{2}}\\ \frac{(c\lambda^{2}-a\eta^{2})b\gamma }{c\lambda^{2}}\\ -b \end{array}\right)$$

we have that

4.33 $$\begin{array}{rl} V(\pm \xi_{1})\;e^{\pm \beta_{1}x} & =\left(\begin{array}{c} \pm \xi_{1}\\ 1 \end{array}\right)e^{\pm \beta_{1}x}=\Phi_{M}(x)a(\pm \beta_{1},\beta_{2})\\ \;\text{and}\;\;V(\pm \xi_{2})\;e^{\pm \beta_{2}x} & =\left(\begin{array}{c} \pm \xi_{2}\\ 1 \end{array}\right)e^{\pm \beta_{2}x}=\Phi_{M}(x)a(\pm \beta_{2},\beta_{1}), \end{array}\}$$

where *ξ*=−*aβ*/(*c*λ^2^−*aβ*^2^).

The characterization of modal waves as the superposition of four exponential waves (4.31) and (4.33) can be written in terms of the matrix basis *Φ*_*M*_(*x*) as

4.34 $$\text{v}(t,x)=e^{\lambda t}(c_{1}^{-}\Phi_{M}(x)a(\beta_{1},\beta_{2})+c_{2}^{-}\Phi_{M}(x)a(\beta_{2},\beta_{1})+c_{1}^{+}\Phi_{M}(x)a(-\beta_{1},\beta_{2})+c_{2}^{+}\Phi_{M}(x)a(-\beta_{2},\beta_{1}))$$

with the advantage that this representation, through a limit procedure, does not require to change the solution basis as is done in the literature when *β*_1_=0. This is clearer when keeping the definition of *Φ*_*M*_(*x*) in terms of the basis generated by *d*(*x*) or using (4.26), that is,

$$\begin{array}{rl} e^{\pm \beta_{1}x} & =ab(\mp \beta_{1}\beta_{2}^{2}d(x)-\beta_{2}^{2}d^{\prime }(x)+(\pm )\beta_{1}d^{\prime \prime }(x)+d^{\prime \prime \prime }(x)),\\ e^{\pm \beta_{2}x} & =ab(\mp \beta_{2}\beta_{1}^{2}d(x)-\beta_{1}^{2}d^{\prime }(x)+(\pm )\beta_{2}d^{\prime \prime }(x)+d^{\prime \prime \prime }(x)). \end{array}$$

## 5. The classes of plane and modal waves

Plane and modal waves are not the same class of solutions. It is possible to exhibit a plane wave that is not a modal wave. For instance, by substituting in (3.4) the constant vectors

$$c_{1}=\left(\begin{array}{c} c\lambda^{2}\\ 0 \end{array}\right),\;c_{2}=\left(\begin{array}{c} 0\\ \frac{c\lambda^{2}(a+e\lambda^{2}-b\beta^{2})}{a\beta } \end{array}\right)$$

with *α*=1 and λ and *β* such that *P*(1)=0, we have the solution

5.1 $$\text{v}(t,x)=\left(\begin{array}{c} \lambda t+\beta x\\ \beta \end{array}\right),$$

which is a plane wave but not a modal wave once modal waves as defined in (4.1) are not linear in *t*.

When considering plane waves **v**(*t*,*x*)=**V**(λ*t*+*βx*), the argument *s*=λ*t*+*βx* is set up with λ and *β* satisfying the equation *P*(*η*)=0 given in (3.7). The roots of (3.7) are *η*=0,0,*α*,−*α* with *η*=*α* denoting the root defined in (3.8). In particular, exponential waves are plane waves for which λ and *β* satisfy the dispersion equation *Q*(λ,*β*)=*b*_0_+*b*_2_=0 given in (3.25). This implies that *α*=1.

For modal waves **v**(*t*,*x*)=e^λ*t*^**w**(*x*), the obtention of the spatial amplitude **w**(*x*) involves the roots *z* of the characteristic equation (4.13). This later is just *b*_0_(λ,*z*)+*b*_2_(λ,*z*)=0 with *b*_0_,*b*_2_ being the coefficients given in (3.7). Therefore, for a comparison of modal waves with plane waves, we must assume that we take into account plane waves with *α*=1, that is, *P*(*η*)=0 having the double root *η*=0 and the simple roots *η*=±1. This latter condition means that the values λ,*β* satisfying the dispersion equation for exponential waves are related with the values λ,*z*(λ) of modal waves, where *z*(λ) is a root of the characteristic equation *Q*(*z*)=0 given in (4.13). When the roots *z*=±*β*_1_,±*β*_2_ of (4.13) are simple, the decomposition of modal waves (4.31) as superposition of exponential waves given in (4.31) or (4.34) shows that a modal wave will involve four plane waves that are of exponential type with the parameter *α*=1.

The critical frequencies λ=0 and λ=λ_c_ occur for both plane and modal waves. The case λ=0 corresponds to a static case with *α*=0 for plane waves and *ϵ*=*δ*=0 in modal waves. For these values, the scalar wave-generating functions *d*_P_(*s*) and *d*_*M*_(*x*) will become cubic polynomials in *x*. The case λ=λ_c_ implies that *α* is arbitrary for plane waves and for *α*=1 we shall have four plane waves that correspond to the roots *β*=0,0,±i*δ* of the characteristic polynomial *Q*(*z*)=0 of modal waves. The case *β*=0, being a double root, is worked out with the Liouville process. This latter will introduce waves with linear variation in *x*.

From the above discussion, we can consider that the class of solutions formed by finite superposition of plane waves contains all modal waves once each modal wave is the superposition of four exponential waves (4.31). Also, the class of exponential wave solutions can be included within the plane or modal waves class of solutions. Moreover, when the outgoing and ingoing evanescent waves collapse, that is, become stationary, it is an indication that we are at a critical frequency that could be treated by the Liouville limit technique.

## 6. Non-local models

The use of continuum theory in carbon nanotubes and atomic force microscopy has been modified in order to include several effects that arise when dealing with small scales and solid–fluid interaction problems [1,15,21,22], among others. In mathematical terms, this amounts to modifying the matrices **M** and **K** in the Timoshenko model (2.1). In what follows, we shall illustrate the methodology with the non-local Timoshenko model [23]

6.1 $$\text{M}\ddot{\text{v}}(t,x)+K\text{v}(t,x)=\text{0},$$

where M=**M**+**M**_*NL*_ and K=**K** with **M** and **K** the matrix coefficient of the Timoshenko model given in (2.2) and

6.2 $${\text{M}}_{NL}=\left(\begin{array}{cc} -{\left(e_{0}a_{0}\right)}^{2}\rho A\frac{\partial^{2}}{\partial x^{2}} & 0\\ 0 & -{\left(e_{0}a_{0}\right)}^{2}\rho I\frac{\partial^{2}}{\partial x^{2}} \end{array}\right).$$

By substituting, *u*(*t*,*x*)=U(λ*t*+*βx*), *ψ*(*t*,*x*)=***Ψ***(λ*t*+*βx*), in (6.1) we have the fourth-order matrix ordinary differential equation

6.3 $${\text{N}}_{P}{\text{V}}^{\left(iv\right)}(s)+{\text{M}}_{P}\text{V}{\;}^{\prime \prime }(s)+{\text{C}}_{P}\text{V}{\;}^{\prime }(s)+{\text{K}}_{P}\text{V}(s)=0,$$

with coefficients

$$\begin{array}{rl} {\text{N}}_{P} & =\left(\begin{array}{cc} -\rho A{\left(e_{0}a_{0}\right)}^{2}\beta^{2}\lambda^{2} & 0\\ 0 & -\rho I{\left(e_{0}a_{0}\right)}^{2}\lambda^{2}\beta^{2} \end{array}\right),\;{\text{M}}_{P}=\left(\begin{array}{cc} \rho A\lambda^{2}-\kappa GA\beta^{2} & 0\\ 0 & \rho I\lambda^{2}-EI\beta^{2} \end{array}\right)\\ {\text{C}}_{P} & =\left(\begin{array}{cc} 0 & \kappa GA\beta \\ -\kappa GA\beta & 0 \end{array}\right)\;{\text{K}}_{P}=\left(\begin{array}{cc} 0 & 0\\ 0 & \kappa GA \end{array}\right)\;\text{V}(s)=\left(\begin{array}{c} U(s)\\ \Psi (s) \end{array}\right) \end{array}$$

and with respect to the plane wave phase *s*=λ*t*+*βx*. As before, we can write the general solution of equation (6.3) as

6.4 $$\text{V}(s)={\text{h}}_{P}(s){\text{c}}_{1}+{\text{h}}_{P}^{\prime }(s){\text{c}}_{2}+{\text{h}}_{P}^{\prime \prime }(s){\text{c}}_{3}+{\text{h}}_{P}^{\prime \prime \prime }(s){\text{c}}_{4}=\Phi_{P}\text{c},$$

where **h**_P_(*s*) is the fundamental 2×2 matrix solution satisfying the initial-value problem

6.5 $$\begin{array}{rl} & {\text{N}}_{P}{\text{h}}_{P}^{\left(iv\right)}(s)+{\text{M}}_{P}{\text{h}}_{P}{\;}^{\prime \prime }(s)+{\text{C}}_{P}{\text{h}}_{P}{\;}^{\prime }(s)+{\text{K}}_{P}{\text{h}}_{P}(s)=\text{0}\\ \;\text{and}\;\; & {\text{h}}_{P}(0)=\text{0},\;{\text{h}}_{P}^{\prime }(0)=\text{0},\;{\text{h}}_{P}^{\prime \prime }(0)=\text{0},\;{\text{N}}_{P}{\text{h}}_{P}^{\prime \prime \prime }(0)=\text{I}, \end{array}\}$$

and generates the 8×2 matrix basis *Φ*_P_=(**h**_P_(*s*) **h**′_P_(*s*) **h**′′_P_(*s*) **h**′′′_P_(*s*)).

The matrix solution **h**_P_(*s*) is now given in closed form [6] as

6.6 $${\text{h}}_{P}(s)=\left(\begin{array}{cc} ad_{P}(s)+(e\lambda^{2}-b\beta^{2})d_{P}^{\prime \prime }(s)-{\left(e_{0}a_{0}\right)}^{2}\beta^{2}\lambda^{2}ed_{P}^{iv}(s) & -\beta d_{P}^{\prime }(s)a\\ \beta d_{P}^{\prime }(s)a & \left(c\lambda^{2}-a\beta^{2})d_{P}^{\prime \prime }(s)-c{\left(e_{0}a_{0}\right)}^{2}\beta^{2}\lambda^{2}d_{P}^{\left(iv\right)}(s\right) \end{array}\right),$$

where *d*_P_(*s*) is the scalar non-local plane wave generator function. This function satisfies now the eighth-order scalar initial value problem

6.7 $$\begin{array}{rl} & b_{0}d_{P}^{\left(viii\right)}(s)+b_{2}d_{P}^{\left(vi\right)}(s)+b_{4}d_{P}^{\left(iv\right)}(s)+b_{6}d_{P}^{\prime \prime }(s)=0\\ \;\text{and}\;\; & b_{0}d_{P}^{\left(8-k\right)}(0)=\delta_{1k},\;k=1,2,\ldots ,8, \end{array}\}$$

where $\det(\eta^{4}{\text{N}}_{P}+\eta^{2}{\text{M}}_{P}+\eta {\text{C}}_{P}+{\text{K}}_{P})=\sum_{i=0}^{8}b_{i}\eta^{8-i}=0$.

Exponential solutions, **v**(*t*,*x*)=e^λ*t*+*βx*^*V* , can be found by solving algebraic eigenvalue problem for the eigenvector *V* that leads to a dispersion equation. Wave solutions of modal type **v**(*t*,*x*)=e^λ*t*^**w**(*x*) for the non-local model are found by solving the second-order differential equation

6.8 $$M{\text{w}}^{\prime \prime }(x)+C{\text{w}}^{\prime }(x)+K\text{w}(x)=\text{0},$$

with

$$\begin{array}{rl} M & =\left(\begin{array}{cc} -a-c{\left(e_{0}a_{0}\right)}^{2}\lambda^{2} & 0\\ 0 & -b-e{\left(e_{0}a_{0}\right)}^{2}\lambda^{2} \end{array}\right),\\ C & =\left(\begin{array}{cc} 0 & a\\ -a & 0 \end{array}\right),\;K=\left(\begin{array}{cc} c\lambda^{2} & 0\\ 0 & e\lambda^{2}+a \end{array}\right). \end{array}$$

The general solution is given by

$$\text{w}(x)=\text{h}(x)a_{1}+{\text{h}}^{\prime }(x)a_{2},$$

where

$$\text{h}(x)=\left(\begin{array}{cc} -bd^{\prime \prime }(x)+(a+e\lambda^{2})d(x)-e{\left(e_{0}a_{0}\right)}^{2}\lambda^{2}d^{\prime \prime }(x) & -ad^{\prime }(x)\\ ad^{\prime }(x) & -ad^{\prime \prime }(x)+c\lambda^{2}d(x)-c{\left(e_{0}a_{0}\right)}^{2}\lambda^{2}d^{\prime \prime }(x) \end{array}\right),$$

and *d*(*x*) satisfies the initial value problem

6.9 $$\begin{array}{rl} & b_{0}d^{\left(iv\right)}(x)+b_{2}d^{\prime \prime }(x)+b_{4}d(x)=0\\ \;\text{and}\;\; & d(0)=0,\;d^{\prime }(0)=0,\;d^{\prime \prime }(0)=0,\;b_{0}d^{\prime \prime \prime }(0)=1, \end{array}\}$$

where *b*_0_, *b*_2_ and *b*_4_ are the coefficients for non-local case, that is, obtained from characteristic problem $\det(\beta^{2}M+\beta C+K)=b_{0}\beta^{4}+b_{2}\beta^{2}+b_{4}=0$, by now depending upon the non-local parameter *e*_0_*a*_0_.

We observe that the above approach can be also applied in model given in [21], that considers K=**K**+**K**_NLaxial_+**K**_NLelastic,_ where

6.10 $$\begin{array}{rl} {\text{K}}_{NL\mathrm{axial}} & =\left(\begin{array}{cc} {\left(e_{0}a_{0}\right)}^{2}N_{x}\frac{\partial^{4}}{\partial x^{4}}-N_{x}\frac{\partial^{2}}{\partial x^{2}} & 0\\ 0 & 0 \end{array}\right)\\ \;\text{and}\;\;{\text{K}}_{NL\mathrm{elastic}} & =\left(\begin{array}{cc} -k_{w}{\left(e_{0}a_{0}\right)}^{2}\frac{\partial^{2}}{\partial x^{2}}+k_{w} & 0\\ 0 & 0 \end{array}\right), \end{array}\}$$

that also leads to fourth-order matrix ordinary differential equation.

## 7. Wave analysis with a Timoshenko beam having a crack

Wave techniques have been employed by several authors in theoretical and experimental methods for damage detection and localization of cracks and other imperfections in structures [24–26], among others. When a wave encounters a defect, it will be subject to reflection and transmission. This phenomenon can provide information about the damage location and size once the existence of cracks can reduce the stiffness of a structure and result in changes of structural dynamic behaviour.

**Figure 3.**
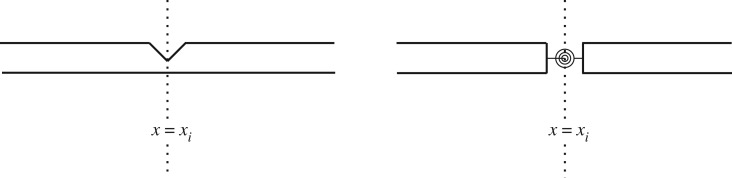
Beam with open crack modelled as rotational spring.

Here, we shall employ the characterization of modal waves (4.34) for discussing the behaviour of a Timoshenko beam with a localized transverse open crack at the point *x*=*x*_*i*_ as shown in figure 3. The crack problem has been modelled by several authors as two segments connected by a massless rotational spring with sectional flexibility spring or with general elastic restraints [27,28].

For a double span with an elastic connection, compatibility conditions are imposed at the location before and after the crack for the displacement, rotation, bending moment and shear force, that is

7.1 $$u_{i}(t,x_{i}^{-})=u_{i+1}(t,x_{i}^{+}),\;\psi_{i}^{\prime }(t,x_{i}^{-})=\psi_{i+1}^{\prime }(t,x_{i}^{+})$$

and

7.2 $$\begin{array}{rl} a\left(\frac{\partial u_{i}}{\partial x}(x_{i}^{-})-\psi_{i}(t,x_{i}^{-})\right) & =a\left(\frac{\partial u_{i+1}}{\partial x}(x_{i}^{+})-\psi_{i+1}(t,x_{i}^{+})\right) \end{array}$$

7.3 $$\begin{array}{rl} \frac{\partial u_{i+1}}{\partial x}(t,x_{i}^{+})-\frac{\partial u_{i}}{\partial x}(t,x_{i}^{-}) & =\theta L\frac{\partial \psi_{i+1}}{\partial x}(t,x_{i}^{+}), \end{array}$$

where *L* is the length of beam, *θ* is the non-dimensional crack sectional flexibility [25],

7.4 $$\theta =6\pi {\bar{\gamma }}^{2}f_{D}(\bar{\gamma })\left(\frac{H}{L}\right)$$

with

7.5 $$f_{D}(\bar{\gamma })=0.6384-1.035\bar{\gamma }+3.7201{\bar{\gamma }}^{2}-5.1773{\bar{\gamma }}^{3}+7.553{\bar{\gamma }}^{4}-7.332{\bar{\gamma }}^{5}+2.4909{\bar{\gamma }}^{6},$$

depending upon a non-dimensional crack–depth ratio $\bar{\gamma }=\tilde{a}/H$, $\tilde{a}$ being the depth of the crack and *H* is the height of beam. Here, $x_{i}^{-}$ and $x_{i}^{+}$ refer to the position immediately at the left and right of *x*_*i*_, respectively.

From a physical point of view, a wave solution that is incident upon the crack localized at *x*=*x*_*i*_ is to be considered reflected and transmitted. Thus, we have the decomposition

7.6 $${\text{v}}_{i}(t,x)={\text{v}}_{i}^{+}(t,x)+{\text{v}}_{i}^{-}(t,x),$$

where

$$\begin{array}{rl} {\text{v}}_{i}^{+} & =c_{1}^{+}\Phi_{M}(x)a(-\beta_{1},\beta_{2})+c_{2}^{+}\Phi_{M}(x)a(-\beta_{2},\beta_{1}),\\ {\text{v}}_{i}^{-} & =c_{1}^{-}\Phi_{M}(x)a(\beta_{1},\beta_{2})+c_{2}^{-}\Phi_{M}(x)a(\beta_{2},\beta_{1}) \end{array}$$

are identified as the incident and reflected waves, respectively. The transmitted modal wave at *x*=*x*_*i*_ is ${\text{v}}_{i+1}(t,x)={\text{v}}_{i+1}^{+}(t,x),$ where

$${\text{v}}_{i+1}^{+}=b_{1}^{+}\Phi_{M}(x)a(-\beta_{1},\beta_{2})+b_{2}^{+}\Phi_{M}(x)a(-\beta_{2},\beta_{1})$$

has unknown amplitudes $b_{1}^{+}$ and $b_{2}^{+}$.

For devices involving spatial rates and time external excitation or a time-forcing boundary condition term, the compatibility conditions can be written in a general form as

7.7 $$C_{1,i}V_{i}(t,x_{i}^{-})=C_{2,i}V_{i+1}(t,x_{i}^{+})+N_{i},$$

where

$$C_{1,i}=\left(\begin{array}{cc} C_{11}^{i} & C_{12}^{i}\\ C_{21}^{i} & C_{22}^{i} \end{array}\right),\;C_{2,i}=\left(\begin{array}{cc} \tilde{C_{11}^{i}} & \tilde{C_{12}^{i}}\\ \tilde{C_{21}^{i}} & \tilde{C_{22}^{i}} \end{array}\right)$$

with $C_{i,j}^{i}$ and $\tilde{C_{i,j}^{i}}$ for *i*,*j*=1,2 are matrix 2×2 and

7.8 $$V_{j}(t,x)=\left(\begin{array}{c} {\text{v}}_{j}\\ {\left({\text{v}}_{j}\right)}_{x} \end{array}\right)=\left(\begin{array}{c} u_{j}(t,x)\\ \psi_{j}(t,x)\\ u_{j}^{\prime }(t,x)\\ \psi_{j}^{\prime }(t,x) \end{array}\right).$$

Here we shall consider that there are no time inputs such as inertial terms and set $N_{i}$=0.

For a crack simulated as a rotational spring at *x*=*x*_*i*_, the compatibility conditions (7.1)–(7.3) give rise to the matrices

7.9 $$\begin{array}{rl} C_{11}^{i} & =\tilde{C_{11}^{i}}=\left(\begin{array}{cc} 1 & 0\\ 0 & 0 \end{array}\right),\;C_{12}^{i}=\tilde{C_{12}^{i}}=\left(\begin{array}{cc} 0 & 0\\ 0 & 1 \end{array}\right),\;C_{21}^{i}=\tilde{C_{21}^{i}}=\left(\begin{array}{cc} 0 & -a\\ 0 & 0 \end{array}\right)\\ \;\;\text{and}\;\;C_{22}^{i} & =\left(\begin{array}{cc} a & 0\\ -1 & 0 \end{array}\right),\;\tilde{C_{22}^{i}}=\left(\begin{array}{cc} a & 0\\ -1 & \theta L \end{array}\right), \end{array}\}$$

By substituting in (7.7) the solutions before and after the crack, it turns out the system

7.10 $$\left(\begin{array}{cccc} A_{3} & A_{4} & -A_{1} & -A_{2}\\ B_{1} & B_{2} & -B_{5} & -B_{6} \end{array}\right)\left(\begin{array}{c} c_{1}^{-}\\ c_{2}^{-}\\ b_{1}^{+}\\ b_{2}^{+} \end{array}\right)=\left(\begin{array}{c} -A_{1}c_{1}^{+}-A_{2}c_{2}^{+}\\ -B_{1}c_{1}^{+}-B_{2}c_{2}^{+} \end{array}\right),$$

for determining the unknown amplitudes $c_{1}^{-}$, $c_{2}^{-}$, $b_{1}^{+}$ and $b_{2}^{+}$. Here, where for simplicity *x*_*i*_=0, we have the matrix coefficients

$$A_{1}=A(\beta_{1},\beta_{2}),\;A_{2}=A(\beta_{2},\beta_{1}),\;A_{3}=A(-\beta_{1},\beta_{2}),\;A_{4}=A(-\beta_{2},\beta_{1})$$

and

$$B_{1}=B_{3}=B(\beta_{2}),\;B_{2}=B_{4}=B(\beta_{1}),\;B_{5}=B_{1}+B(\beta_{1}),\;B_{6}=B_{2}+B(\beta_{2})$$

with

7.11 $$A(\gamma ,\eta )=\left(\begin{array}{c} \frac{(c\lambda^{2}-a\eta^{2})\gamma b}{ca\lambda^{2}}\\ -\gamma \end{array}\right),\;B(\beta )=\left(\begin{array}{c} \frac{b(\beta^{2}a-c\lambda^{2})}{a}\\ -\frac{ab\beta^{2}-bc\lambda^{2}+a^{2}}{a^{2}} \end{array}\right),\;B(\beta )=\left(\begin{array}{c} 0\\ -\theta L\beta \end{array}\right).$$

By solving the system (7.10), we have

$$c^{-}=\text{R}c^{+},\;b^{+}=\text{T}c^{+},$$

where **R** and **T** are reflection and transmission matrices given by

$$\begin{array}{rl} \text{R} & =\mu \left(\begin{array}{cc} -\beta_{1}c\lambda^{2}+\beta_{1}^{3}a & \beta_{2}\beta_{1}^{2}a-\beta_{2}c\lambda^{2}\\ -\beta_{1}\beta_{2}^{2}a+\beta_{1}c\lambda^{2} & -\beta_{2}^{3}a+\beta_{2}c\lambda^{2} \end{array}\right),\;\text{T}=\text{I}-\text{R},\\ \mu & =\frac{L\theta }{-\theta L(\beta_{2}(\beta_{2}^{2}a-c\lambda^{2})+\beta_{1}(-\beta_{1}^{2}a+c\lambda^{2}))-2a(\beta_{2}^{2}-\beta_{1}^{2})}. \end{array}$$

The same matrix procedure can be applied to the case of finding natural frequencies with boundary conditions or intermediate devices [3].

**Figure 4.**
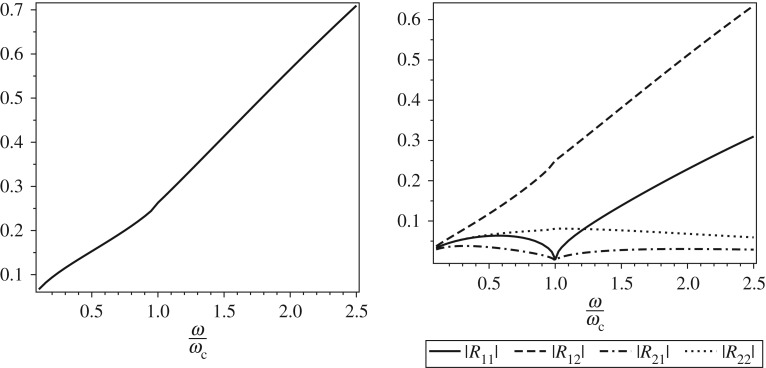
Norm matrix and modulus of coordinates of the matrix **R**.

Simulations have been performed for a single-sided crack with depth $\tilde{a}=3.5$ mm, width *B*=10 mm, height *H*=10 mm and parameters *E*=2.07×10^11^ N m^−2^, *ρ*=7860 kg m^−3^, *ν*=0.3 and *γ*=0.35, as given in [26]. The results with the Euclidian matrix norm ∥**R**∥_2_ of the reflection matrix and the modulus of each of its components are presented in figure 4. It is observed that the value norm ∥**R**∥_2_ is more influenced by |**R**_12_| which, in turn, influences the amplitude of reflected wave component with e^*β*_1_*x*^. The behaviour of Euclidian norm of the transmitted matrix ∥**T**∥_2_ is more influenced by the diagonal components of reflection matrix.

**Figure 5.**
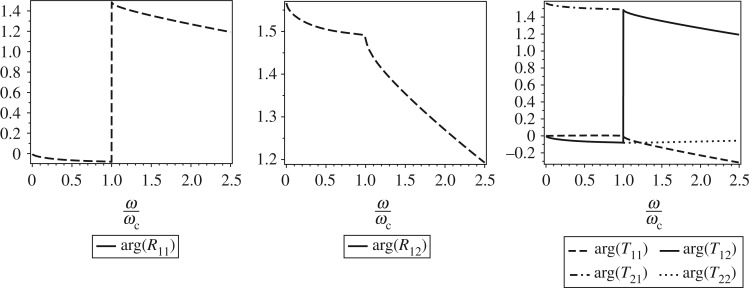
Argument of coordinates of the matrices **R** and **T**.

The arguments of the components of **R** and **T** are presented in figure 5. For low frequencies, the arguments arg(**R**_12_) and arg(**T**_21_) are more significant and represent the phase of reflected and transmitted wave, respectively. Simulations show that for low frequencies arg(*R*_21_) is close to arg(*R*_11_) and arg(*R*_22_) is close to arg(*R*_12_). For high frequencies, arg(**R**_*ij*_) and arg(**T**_*ij*_), *i*,*j*=1,2, tend to constants values.

## 8. Boundary conditions

**Figure 6.**
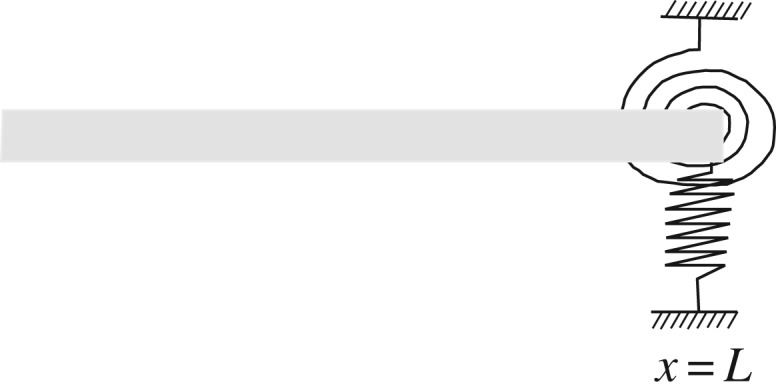
Non-classical boundary conditions at *x*=*L*.

When we consider an incident wave at the end of a finite beam, there is a reflected wave to be determined from the incident wave and the boundary conditions. Elastic translational and rotational devices at the end of a beam are non-classical boundary conditions that through a limit process can include the classical boundary conditions such as supported, free and clamped beams [29]. In [3], such case was discussed by using exponential waves away from critical frequencies. Here, we shall introduce an ingoing and outgoing decomposition of the wave modal matrix basis *Φ*_*M*_(*x*) that can be used also for critical frequencies. For the beam, in figure 6, the physical boundary conditions are

8.1 $$\begin{array}{rl} -V & =-a\left(\frac{\partial u}{\partial x}(t,L)-\psi (t,L)\right)=K_{T}u(t,L)+K_{\mathrm{TR}}\psi (t,L)\\ \;\text{and}\;\;M & =-b\frac{\partial \psi }{\partial x}(t,L)=K_{R}\psi (t,L)+K_{\mathrm{RT}}u(t,L), \end{array}$$

where *V* (*t*,*x*) is shear force and *M*(*t*,*x*) is bending moment, *K*_T_ and *K*_*R*_ are translational and rotational stiffnesses and *K*_TR_, *K*_RT_ are coupling translational and rotational stiffnesses. These boundary conditions can be written in matrix form as

8.2 $${\text{A}}_{1}\text{v}(t,L)+{\text{A}}_{2}{\text{v}}_{x}(t,L)={\text{B}}_{1}\text{v}(t,L)+{\text{B}}_{2}{\text{v}}_{x}(t,L)$$

where

8.3 $${\text{A}}_{1}=\left(\begin{array}{cc} 0 & a\\ 0 & 0 \end{array}\right),\;{\text{A}}_{2}=\left(\begin{array}{cc} -a & 0\\ 0 & -b \end{array}\right),\;{\text{B}}_{1}=\left(\begin{array}{cc} K_{T} & K_{\mathrm{TR}}\\ K_{\mathrm{RT}} & K_{R} \end{array}\right),\;{\text{B}}_{2}=\left(\begin{array}{cc} 0 & 0\\ 0 & 0 \end{array}\right).$$

Following the matrix treatment given for compatibility conditions (7.7), we write the boundary conditions in the compact form

8.4 AV(t,L)=BV(t,L)

where **A**, **B** and given in block form as

8.5 $$\text{A}=({\text{A}}_{1}\;{\text{A}}_{2}),\;\text{B}=({\text{B}}_{1}\;{\text{B}}_{2}),\;V(t,L)=\left(\begin{array}{c} \text{v}(t,L)\\ {\text{v}}_{x}(t,L) \end{array}\right).$$

The wave decomposition (7.6) of a general modal wave

8.6 $$\text{v}(t,x)=e^{\lambda t}\Phi_{M}(x)a={\text{v}}^{+}(t,x)+{\text{v}}^{-}(t,x)$$

can be further written in terms of a decomposed scalar modal wave generator function *d*(*x*) given in (4.14). We have that as

8.7 $$d(x)=d^{+}(x)+d^{-}(x),$$

where

8.8 $$d^{+}(x)=\frac{1}{2}\frac{-\beta_{2}\;e^{-\beta_{1}x}+\beta_{1}\;e^{-\beta_{2}x}}{ab(-\beta_{2}^{2}+\beta_{1}^{2})\beta_{1}\beta_{2}},\;d^{-}(x)=\frac{1}{2}\frac{\beta_{2}\;e^{\beta_{1}x}-\beta_{1}\;e^{\beta_{2}x}}{ab(-\beta_{2}^{2}+\beta_{1}^{2})\beta_{1}\beta_{2}},$$

this decomposition allows to write $\Phi_{M}(x)=\Phi_{M}^{+}(x)+\Phi_{M}^{-}(x)$ where according with (4.29)

8.9 $$\Phi_{M}^{+}(x)=e^{-\beta_{1}x}\Psi_{1}(-\beta_{1})+e^{-\beta_{2}x}\Psi_{2}(-\beta_{2}),\;\Phi_{M}^{-}(x)=e^{\beta_{1}x}\Psi_{1}(\beta_{1})+e^{\beta_{2}x}\Psi_{2}(\beta_{2}),$$

being *Ψ*_*i*_(*β*), *i*=1,2, given in (4.30) resulting in

8.10 $$\text{v}(t,x)={\text{v}}^{+}(t,x)+{\text{v}}^{-}(t,x)=e^{\lambda t}(\Phi_{M}^{+}(x)a+\Phi_{M}^{-}(x)a),$$

where ${\text{v}}^{+}(t,x)=e^{\lambda t}\Phi_{M}^{+}(x)a$ and ${\text{v}}^{-}(t,x)=e^{\lambda t}\Phi_{M}^{-}(x)a$.

By assuming that we know the incident wave at the boundary, then the 2×1 wave value $\text{ b}=\Phi_{M}^{+}(L)a$ at the boundary is known and imposes two restrictions for obtaining the 4×1 vector a. Thus, the system $\Phi_{M}^{+}(L)a=\text{b}$ will allow to write

8.11 $$a=\Lambda \tilde{a}+\Sigma$$

with

8.12 $$\Lambda =\left(\begin{array}{cc} \alpha_{1} & \alpha_{2}\\ \alpha_{3} & \alpha_{4}\\ 1 & 0\\ 0 & 1 \end{array}\right),\;\Sigma =\left(\begin{array}{c} \sigma_{1}\\ \sigma_{2}\\ 0\\ 0 \end{array}\right),\;\tilde{a}=\left(\begin{array}{c} a_{12}\\ a_{22} \end{array}\right),$$

where *a*_11_,*a*_21_ depend linearly on *a*_11_ and *a*_22_, that is,

$$\begin{array}{r} a_{11}=a_{11}(a_{12},a_{22})=\alpha_{1}a_{12}+\alpha_{2}a_{22}+\sigma_{1},\\ a_{21}=a_{21}(a_{12},a_{22})=\alpha_{3}a_{12}+\alpha_{4}a_{22}+\sigma_{2}. \end{array}$$

The substitution of the wave matrix decomposition (8.10) in (8.4) leads to

8.13 $$[({\text{A}}_{1}-{\text{B}}_{1})(\Phi_{M}^{+}(L)+\Phi_{M}^{-}(L))+({\text{A}}_{2}-{\text{B}}_{2})(\Phi_{M}^{+}{\left(L\right)}_{x}+\Phi_{M}^{-}{\left(L\right)}_{x})]a=\text{0}.$$

By using (8.11), we have to solve the 2×2 linear system

8.14 $${\text{M}}_{a}\tilde{a}=\text{G},$$

where

$$\begin{array}{rl} {\text{M}}_{a} & =[({\text{A}}_{1}-{\text{B}}_{1})(\Phi_{M}^{+}(L)+\Phi_{M}^{-}(L))+({\text{A}}_{2}-{\text{B}}_{2})(\Phi_{M}^{+}{\left(L\right)}_{x}+\Phi_{M}^{-}{\left(L\right)}_{x})]\Lambda ,\\ \text{G} & =-[({\text{A}}_{1}-{\text{B}}_{1})(\Phi_{M}^{+}(L)+\Phi_{M}^{-}(L))+({\text{A}}_{2}-{\text{B}}_{2})(\Phi_{M}^{+}{\left(L\right)}_{x}+\Phi_{M}^{-}{\left(L\right)}_{x})]\Sigma . \end{array}$$

Thus, the reflected wave is given by ${\text{v}}^{-}(t,x)=e^{\lambda t}\Phi_{M}^{-}(x)a=e^{\lambda t}\Phi_{M}^{-}(x)(\Lambda \tilde{a}+\sigma )$. The same procedure will be followed for the case of boundary conditions at the other end *x*=0.

## 9. Conclusion

Plane waves are the simplest solutions of the Timoshenko beam equations that assume a constant value through points on a line that involves a frequency and a wavenumber. These parameters can be real or complex numbers in order to cope with the study of complicating effects such as damping, discontinuities, dispersive and complex materials, attached devices or obstacles, among others, as well as harmonic behaviour or normal property that is not often feasible [4,17]. We have completely characterized them in terms of matrix basis involving the fundamental solution of a fourth-order scalar differential equation. Plane waves involve exponential or linear behaviour and they have proportional components only when they propagate in two defined directions whose amplitude is exponential or linear. Modal waves include as particular cases standing waves or waves with the normal property according to boundary conditions. They have been completely characterized in terms of a matrix basis that involves the fundamental solution of a scalar fourth-order differential equation. They can be written as the superposition of four plane waves and their class include the waves with exponential profile. This methodology can be extended to non-local Timoshenko models.

Reflected and transmitted modal waves for a crack problem and an incident wave at a boundary point of the beam have been characterized by using a decomposition of ingoing and outgoing terms of the modal matrix basis *Φ*_*M*_(*x*). Simulations for the crack problem illustrate the influence of certain components before and after the critical frequency value.

## Data accessibility

Does not apply.

## References

[1] Gopalakrishnan S, Narendar S 2013 *Wave propagation in nanostructures: nonlocal continuum mechanics formulations*. Berlin, Germany: Springer.

[2] Iglesias AM, Wicks AL, Schwartz T 2009 Interactions of modal analysis and wave propagation using Timoshenko beam model. *Proc. IMAC-XXVII, Orlando, FL, 9–12 February*, Society for Experimental Mechanics Inc.

[3] Mei C, Mace BR 2005 Wave reflection and transmission in Timoshenko beams and wave analysis of Timoshenko beam structures. *J. Vib. Acoust.* 127, 382–394. (doi:10.1115/1.1924647)

[4] Kang B 2011 Exact transfer function analysis of distributed parameter systems by wave propagation techniques. In *Recent advances in vibrations analysis* (ed. N Baddour), chap. 1. Rijeka, Croatia: InTech. (doi:10.5772/861)

[5] Ham S, Bathe KJ 2012 A finite element method enriched for wave propagation problems. *Comput. Struct.* 94–95, 1–12. (doi:10.1016/j.compstruc.2012.01.001)

[6] Claeyssen JR, Canahualpa G, Jung C 1999 A direct approach to second-order matrix non-classical vibrating equations. *Appl. Numer. Math.* 30, 65–78. (doi:10.1016/S0168-9274(98)00085-3)

[7] Claeyssen JR, Costa SNJ 2006 Modes for the coupled Timoshenko model with a restrained end. *J. Sound Vib.* 296, 1053–1058. (doi:10.1016/j.jsv.2006.02.025)

[8] Claeyssen JR, Soder R 2003 A dynamical basis for computing the modes of Euler–Bernoulli and Timoshenko beams. *J. Sound Vib.* 259, 986–990. (doi:10.1006/jsvi.2002.5232)

[9] Huang TC 1961 The effect of rotatory inertia and of shear deformation on the frequency and normal mode equations of uniform beams with simple end conditions. *J. Appl. Mech.* 28, 579–584. (doi:10.1115/1.3641787)

[10] Harris JC 2001 *Linear elastic waves*. Cambridge, UK: Cambridge University Press.

[11] Ginsberg JH 2001 *Mechanical and structural vibrations: theory and applications*. New York, NY: John Wiley & Sons, Inc.

[12] Olsson P, Kristensson G 1994 Wave splitting of the Timoshenko beam equation in the time domain. *Z. Angew. Math. Phys.* 45, 1–14. (doi:10.1007/BF00942843)

[13] Carvalho MOM, Zindeluk M 2001 Active control of waves in a Timoshenko beam. *Int. J. Solids Struct.* 38, 1749–1764. (doi:10.1016/S0020-7683(00)00134-7)

[14] Graff KF 1991 *Wave motion in elastic solids*. New York, NY: Dover Publications.

[15] Liu C, Rajapakse RKND 2010 Continuum models incorporating surface energy for static and dynamic response of nanoscale beams. *IEEE Trans. Nanorechnol.* 9, 422–431. (doi:10.1109/TNANO.2009.2034142)

[16] Banks HT, Inman D 1991 On damping mechanisms in beams. *J. Appl. Mech.* 58, 716–723. (doi:10.1115/1.2897253)

[17] Friswell MI, Adhikari S, Lei Y 2007 Non-local finite element analysis of damped beams. *Int. J. Solids Struct.* 44, 7564–7576. (doi:10.1016/j.ijsolstr.2007.04.023)

[18] Claeyssen JR, Tsukazan T, Copetti RD 2013 Nonlocal effects in modal analysis of forced responses with single carbon nanotubes. *Mech. Syst. Signal Process.* 38, 299–311. (doi:10.1016/j.ymssp.2013.01.014)

[19] Chan KT, Wang XQ, So RMC, Reid SR 2001 Superposed standing waves in a Timoshenko beam. *Proc. R. Soc. Lond. A* 458, 83–108. (doi:10.1098/rspa.2001.0855)

[20] van Rensburg NFJ, van der Merwe AJ 2006 Natural frequencies and modes of a Timoshenko beam. *Wave Mot.* 44, 58–69. (doi:10.1016/j.wavemoti.2006.06.008)

[21] Wang YZ, Li FM, Kishimoto K 2012 Effects of axial load and elastic matrix on flexural wave propagation in nanotube with nonlocal Timoshenko beam model. *J. Vib. Acoust.* 134, 031011-1–031011-7. (doi:10.1115/1.4005832)

[22] Wang YZ, Cui HT, Li FM, Kishimoto K 2011 Effects of viscous fluid on wave propagation in carbon nanotubes. *Phys. Lett. A* 375, 2448–2451. (doi:10.1016/j.physleta.2011.05.016)

[23] Lu P, Lee HP, Lu C, Zhang PQ 2007 Application of nonlocal beam models for carbon nanotubes. *Int. J. Solids Struct.* 44, 5289–5300. (doi:10.1016/j.ijsolstr.2006.12.034)

[24] Chondros TG, Dimarogonas AD 1989 Influence of a crack on the dynamic characteristics of structures. *J. Vib. Acoust. Stress Reliab. Des.* 111, 251–6. (doi:10.1115/1.3269849)

[25] Ostachowicz WM, Krawczuk M 1991 Analysis of the effect of cracks on the natural frequencies of a cantilever beam. *J. Sound Vib.* 150, 191–201. (doi:10.1016/0022-460X(91)90615-Q)

[26] Lin HP 2004 Direct and inverse methods on free vibration analysis of simply supported beams with a crack. *Eng. Struct.* 26, 427–436. (doi:10.1016/j.engstruct.2003.10.014)

[27] Yang X, Huang J, Ouyang Y 2016 Bending of Timoshenko beam with effect of crack gap based on equivalent spring model. *Appl. Math. Mech.* 37, 513–528. (doi:10.1007/s10483-016-2042-9)

[28] Lele SP, Maiti SK 2002 Modelling of transverse vibration of short beams for crack detection and measurement of crack extension. *J. Sound Vib.* 257, 559–583. (doi:10.1006/jsvi.2002.5059)

[29] Balachandran B, Magrab EB 2009 *Vibrations*. Toronto, Canada: Cengage Learning.

